# A CYC–RAD–DIV–DRIF interaction likely pre-dates the origin of floral monosymmetry in Lamiales

**DOI:** 10.1186/s13227-021-00187-w

**Published:** 2022-01-29

**Authors:** Aniket Sengupta, Lena C. Hileman

**Affiliations:** 1 Department of Ecology and Evolutionary Biology, University of Kansas, 1200 Sunnyside Avenue, Lawrence, KS 66045 USA; 2 Present Address: St. Albert Hall, 8000 Utopia Pkwy, Room 257, Queens, NY 11439 USA

**Keywords:** CYCLOIDEA, Floral monosymmetry, Genetic program, Lamiales, RADIALIS, Solanales

## Abstract

**Background:**

An outstanding question in evolutionary biology is how genetic interactions defining novel traits evolve. They may evolve either by de novo assembly of previously non-interacting genes or by *en bloc* co-option of interactions from other functions. We tested these hypotheses in the context of a novel phenotype—Lamiales flower monosymmetry—defined by a developmental program that relies on regulatory interaction among *CYCLOIDEA*, *RADIALIS*, *DIVARICATA*, and *DRIF* gene products. In *Antirrhinum majus* (snapdragon), representing Lamiales, we tested whether components of this program likely function beyond their previously known role in petal and stamen development. In *Solanum lycopersicum* (tomato), representing Solanales which diverged from Lamiales before the origin of Lamiales floral monosymmetry, we additionally tested for regulatory interactions in this program.

**Results:**

We found that *RADIALIS*, *DIVARICATA*, and *DRIF* are expressed in snapdragon ovaries and developing fruit, similar to their homologs during tomato fruit development. In addition, we found that a tomato *CYCLOIDEA* ortholog positively regulates a tomato *RADIALIS* ortholog.

**Conclusion:**

Our results provide preliminary support to the hypothesis that the developmental program defining floral monosymmetry in Lamiales was co-opted *en bloc* from a function in carpel development. This expands our understanding of novel trait evolution facilitated by co-option of existing regulatory interactions.

**Supplementary Information:**

The online version contains supplementary material available at 10.1186/s13227-021-00187-w.

## Background

Convergent traits are novel traits (derived characters, apomorphies) that have recurrently evolved across the tree of life. Interestingly, novel traits usually do not evolve by utilizing new genes, but evolve by co-opting existing genes and genetic programs from other functions. For example, compound leaves, a novelty repeatedly derived in many flowering plant lineages, are defined by recruitment of *KNOTTED1-like homeobox* (*KNOX*) genes, a gene family that ancestrally is involved in meristem development [1, 2]. However, gene products do not usually function in isolation but interact with other gene products as a part of genetic programs (pathways or networks) to affect phenotype. Hence, it is likely that any gene co-opted towards defining a novel trait was part of a genetic program in the ancestral species. It is not always evident whether individual gene products defining a novel phenotype were co-opted individually from separate networks and assembled into a new network concurrently with the origin of the novelty (de novo assembly), or whether an existing program and set of genetic interactions was co-opted as a unit (*en bloc* co-option). Few studies have addressed this question [2, reviewed in 3, 4], and mostly in animal systems. In the plant *Asparagus*, suggestive evidence based on expression of genes in the cladodes (which are analogous to leaves) indicates that two genetic programs have been co-opted *en bloc* from leaf to cladode development. First, the program involving KNOTTED1-LIKE HOMEOBOX and ASYMMETRIC LEAVES 1 that defines development of true leaves from meristems [5, reviewed in 6]. Second, the program involving PHABULOSA, REVOLUTA, and miR166, that defines the differentiation of the flattened abaxial–adaxial surfaces of leaves [5, reviewed in 6].

Monosymmetric (bilaterally symmetrical, zygomorphic) flowers are a trait novelty that has evolved at least 130 times from polysymmetric (radially symmetrical, actinomorphic) flowers during the diversification of flowering plants [7]. Monosymmetric flowers have one axis of symmetry that divides the flower into a pair of mirror images; polysymmetric flowers have at least two identical axes. Monosymmetric flowers are often associated with specialized pollination by animals [8, reviewed in 9], and occasionally with wind pollination [10, 11; possibly because Poaceae flowers are densely packed and monosymmetry potentially increases access to the wind]. Transitions to monosymmetry are strongly associated with increased speciation rates [12, 13], consistent with its role as a key morphological innovation, or possibly because the potential for newer pollinators provides ground for species selection [14].

**Fig. 1:**
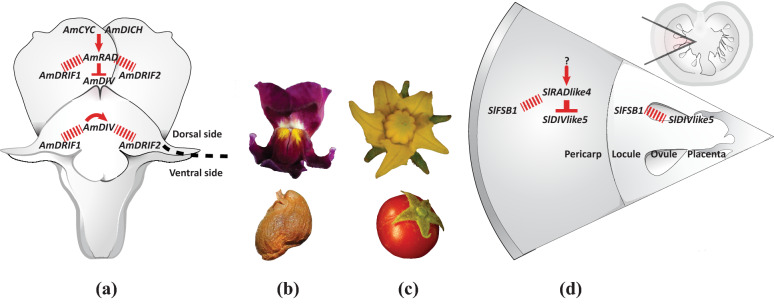
Similar genetic interaction controls flower symmetry in *A. majus* and fruit development in *S. lycopersicum*. **a** Floral monosymmetry in *A. majus* is defined by a CYC–RAD–DIV–DRIF interaction. **b** Flower (top) and fruit (bottom) of *A. majus*. **c** Flower (top) and fruit (bottom) of *S. lycopersicum.* **d** Pericarp development in *S. lycopersicum* is defined by a RAD–DIV–DRIF interaction (*Sl*FSB1 is a DRIF homolog). Red arrow: transcriptional activation of a gene by a transcription factor, red inverted-T: negative regulation of one protein by another, red dashed line: protein–protein interaction

The genetic basis of flower monosymmetry is best understood in the order Lamiales which includes the model species *Antirrhinum majus* (snapdragon). Monosymmetric flowers evolved early during the diversification of Lamiales [7, 15]. Therefore, the lineage leading to *A. majus* has experienced only one shift from poly- to monosymmetry, making this an appropriate system to study the genetic basis of this transition. *Antirrhinum majus* flowers have morphologically distinct dorsal and ventral sides (Fig. 1). Monosymmetry along the dorso-ventral axis in *A. majus* flowers is defined by a competitive interaction involving TCP (TEOSINTE BRANCHED1, CYCLOIDEA, and PROLIFERATING CELL FACTORS) and MYB (first described from an avian myeloblastosis virus) transcription factors. Both *TCP* and *MYB* genes are found as large gene families in flowering plants [16, 17] and play diverse roles beyond flower symmetry patterning, including aspects of vegetative and reproductive development [16, 18, 19].

The dorsal side of an *Antirrhinum* flower, excluding the gynoecium, consists of the dorsal sepal, dorsal portions of the lateral sepals, the dorsal petals, the dorsal portions of the lateral petals, and the dorsal sterile stamen (staminodium) whose development is suppressed early in floral development. The identity of dorsal organs in the petal and stamen whorls is defined by the combined action of two recently duplicated TCP paralogs, CYCLOIDEA (*Am*CYC) and DICHOTOMA (*Am*DICH) [20–23]. These two transcription factors define dorsal flower morphology partly by activating the transcription of a downstream *MYB* gene, *RADIALIS* (*AmRAD*; Fig. 1) [24]. A*m*RAD protein competes with another MYB protein, DIVARICATA (*Am*DIV) which defines ventral petal and stamen whorl morphology. Through this antagonistic interaction, *Am*RAD excludes the ventral flower identity specified by *Am*DIV from the dorsal side of the developing snapdragon flower (Fig. 1). Specifically, *Am*RAD and *Am*DIV compete for interaction with two other MYB-family protein partners called DIV and RAD Interacting Factors 1 and 2 (*Am*DRIF1 and *Am*DRIF2) [24–27] (Fig. 1). *Am*DIV requires protein–protein interaction with *Am*DRIF1&2 to function as a transcription factor to regulate downstream targets (Fig. 1) [27, 28]. In the dorsal flower domain, *Am*RAD outcompetes *Am*DIV for interaction with *Am*DRIF1&2, thereby negatively regulating *Am*DIV function [27].

Evidence strongly supports the hypothesis that *CYC, RAD,* and *DIV* genes and protein interactions are conserved in specifying monosymmetric flower development dating back to a common ancestor early in the diversification of Lamiales [20, 21, 24, 27, 29–38]. This is not surprising; flower monosymmetry is homologous across Lamiales, derived from a monosymmetric ancestor early in Lamiales diversification (although there have been multiple reversals in derived Lamiales lineages) [7, 15]. Whether the CYC–RAD–DIV–DRIF interaction was assembled de novo at the base of Lamiales or was recruited *en bloc* to a role in flower monosymmetry as a pre-assembled unit remains unknown. If the CYC–RAD–DIV–DRIF interaction was recruited as a pre-assembled unit, this would constitute evidence that transitions to floral monosymmetry are facilitated by the presence of an ancestral genetic interaction that can be re-deployed *en bloc* to a novel role in flower development. To test these hypotheses, it is important to determine whether the CYC–RAD–DIV–DRIF interaction has functions beyond flower monosymmetry in Lamiales, and whether this interaction is also present in an outgroup that diverged from the common ancestor of Lamiales before Lamiales flower monosymmetry evolved.

Solanales are the sister order to Lamiales + Vahliaceae [39] and primarily develop polysymmetric flowers. The Solanales model species, tomato (*Solanum lycopersicum*), is an ideal outgroup to study the ancestral function of the CYC–RAD–DIV–DRIF network. There are two major groups in Solanales—Convolvulaceae and Solanaceae. Reconstructing ancestral flower symmetry in Solanaceae has been challenging given that the first diverging lineage has monosymmetric corolla. However, recent research suggests that the ancestral Solanales flower likely had polysymmetric corollae [40]. We attempted to develop virus-induced gene silencing in two species from Convolvulaceae (*Ipomoea lobata* and *I. lacunosa*), but silencing was only effective in early stages of plant development (data not shown). Hence, Convolvulaceae and early diverging Solanaceae (that have monosymmetric flowers) are not ideal for comparative analysis. Given these issues, we selected *S. lycopersicum* as a representative of Solanales for comparative analysis.

Compelling data from studies in *S. lycopersicum* suggest that an RAD–DIV–DRIF interaction plays a role in tomato fruit development by modulating cell size [41]. The RAD component, *Sl*RADlike4 (or fruit SANT/MYB-like 1, *Sl*FSM1), is an ortholog of *Am*RAD [42, 43]. *SlRADlike4* is primarily expressed in the tomato pericarp [Tomato Expression Atlas, 44] and suppresses cell expansion in that tissue [41] by competing with a DIV-like protein (Fig. 1d). The DIV component, *Sl*DIVlike5 (*Sl*MYBI) is not an ortholog, but a paralog, of *Am*DIV [42, 43] (Additional file 1: Fig. S4) and is expressed throughout the developing fruit. Similarly, the DRIF component, Fruit SANT/MYB Binding protein1 (*Sl*FSB1) is also not an ortholog, but a paralog of *Am*DRIF1&2 [27] (Additional file 1: Fig. S3). The surprising similarity of this three-component regulatory interaction (Fig. 1) raises the possibility that the common ancestor of Lamiales and Solanales possessed an RAD–DIV–DRIF module to regulate some aspect of plant development and that this module was re-deployed *en bloc* to a role patterning flower monosymmetry during Lamiales diversification.

The similarity between the RAD–DIV–DRIF module in Solanales and Lamiales can best be explained by two scenarios. One, the RAD–DIV–DRIF evolved independently in Solanales and in Lamiales, and hence the DIV and DRIF components are not orthologous between the RAD–DIV–DRIF interaction reported from Lamiales and Solanales. Alternatively, the RAD–DIV–DRIF interaction evolved before the divergence of Solanales and Lamiales. In the second scenario, the lack of orthology between the *A. majus* and *S. lycopersicum* DIV and DRIF components need not exclude the possibility of the RAD–DIV–DRIF module being homologous. This is because DIV and DRIF proteins are a part of the large protein family of MYB factors making it possible for one DRIF paralog to replace another, or one DIV paralog to replace another, in a genetic interaction, especially if these paralogs have similar biochemical properties. Yeast-two-hybrid assays provide evidence that RAD–DIV–DRIF interactions are not ortholog-specific across seed-plants [45]. Indeed, all three clades of DRIFs (Additional file 1: Fig. S3) have at least one member that has been shown to have a DIV–DRIF and RAD–DRIF interaction that is associated with a biological function, suggesting that a DIV–DRIF interaction is likely ancestral to DRIF proteins in Solanales + Lamiales. The three DRIF clades are Group-1 (which includes SlFSB1 that shows DIV–DRIF interaction in tomato fruits), Group-2A (which includes AmDRIF1), and Group-2B (which includes AmDRIF2).

Therefore, despite the lack of strict orthology between the DIV and DRIF components, the RAD–DIV–DRIF interactions displayed by these paralogs may be identical by decent, inherited by Solanales and Lamiales from a common ancestor. Two neofunctionalization scenarios can explain the lack of orthology between the *A. majus* and *S. lycopersicum* DIV and DRIF components: regular neofunctionalization or neofunctionalization associated with paralog replacement. In the first scenario, multiple, ancestral combinations of RAD–DIV–DRIF interactions with overlapping functions existed, but one interaction was neofunctionalized towards monosymmetry [RAD–DIV–DRIF interactions are not ortholog-specific across seed-plants, at least when tested with yeast-two-hybrids assays, 45]. In the second scenario, a unique, ortholog-specific RAD–DIV–DRIF interaction was present in the common ancestor, was neofunctionalized towards monosymmetry, then modified in one of the daughter lineages (where the RAD, or the DIV–DRIF components were replaced by their paralogs). Paralog replacement, in which one paralog replaces another in a biological function, is a documented phenomenon. For example, the replacement of the synaptic function of Acetylcholinesterase1 by its paralog Acetylcholinesterase2 in Cyclorrhapha flies [46].

Here, we tested whether the genes involved in *A. majus* CYC–RAD–DIV–DRIF interaction are expressed, and hence likely functional, in organs not associated with corolla monosymmetry, especially in carpel and fruit development. We also, determined expression patterns for orthologs of these genes in *S. lycopersicum*. An RAD–DIV–DRIF interaction is already known in *S. lycopersicum* fruit development [41]. In addition, we tested whether a CYC–RAD interaction is present in *S. lycopersicum*, by estimating the changes in the transcription of a *S. lycopersicum RAD* ortholog in a *S. lycopersicum CYC*-downregulated background. We also determined whether presence of predicted TCP/CYC-binding sites in upstream regulatory region of *AmRAD* orthologs is ancestral to Lamiales + Solanales. Our results suggest that a CYC–RAD–DIV–DRIF interaction may be ancestral to Lamiales and Solanales and may have been co-opted *en bloc* to flower monosymmetry from another function, likely carpel/fruit development.

## Results

### Patterns of *AmRAD*, *AmDIV*/*DIV*-*like1* and *AmDRIF1*&*2* expression are consistent with a function in carpel and fruit development

**Fig. 2:**
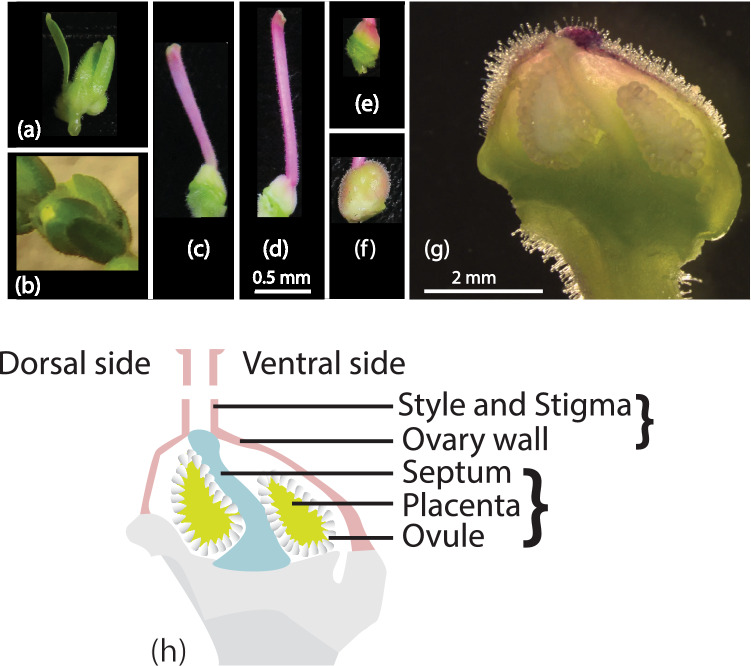
*Antirrhinum majus* reproductive tissues for qRT-PCR. **a** Inflorescence. **b** Flower bud, stage-11. **c** Carpel, stage-13. **d** Carpel, stage-14 (anthesis). **e** Ovary (developing fruit) 7 days after anthesis. **f** Ovary (developing fruit) 11 days after anthesis. **g** Longitudinal section of stage-14 ovary. **h** Thematic representation of carpel; bracketed tissues were sampled together. Scale bars: (**a**–**f**) 0.5 mm, **f** 2 mm

We used quantitative real-time PCR (qRT-PCR) to determine relative expression of *A. majus* flower symmetry genes across stages of carpel and fruit development to assess evidence for RAD–DIV–DRIF function during carpel/fruit development similar to that found in tomato [41]. Expression of these genes in organ primordia has already been tested [20, 21, 24, 26]. Therefore, we tested for expression in later stages of carpel and fruit development (carpel and fruit images in Fig. 2). The genes *AmCYC*, *AmDICH*, *AmRAD*, *AmDIV*, *AmDRIF1*, and *AmDRIF2* are involved in defining flower monosymmetry in *A. majus.* The gene *AmDIV-like1*, a close paralog of *AmDIV*, has not been implicated in the control of flower symmetry, but is important for understanding the ancestral expression and function of its paralog, *AmDIV*.

**Fig. 3:**
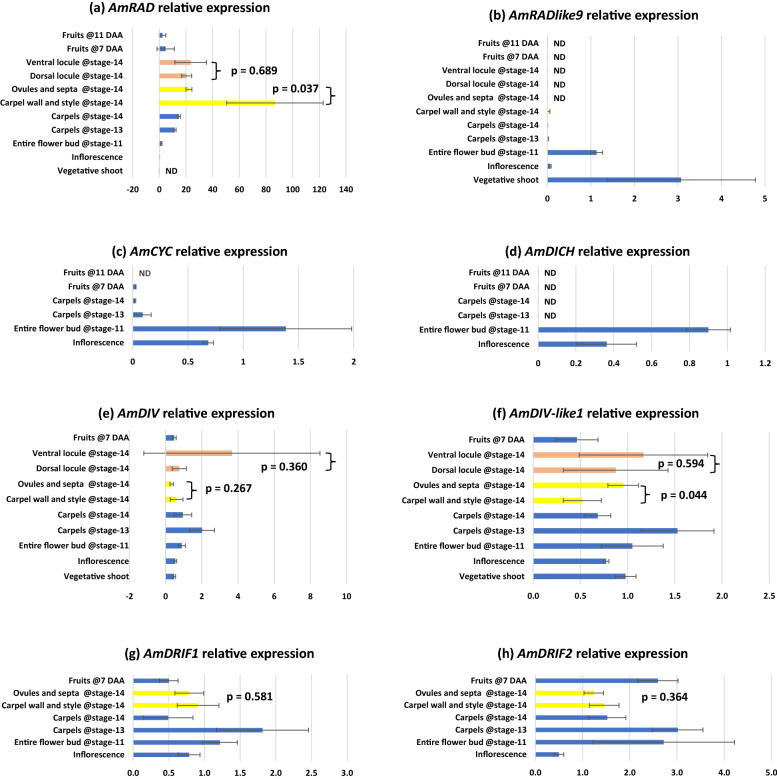
Relative expression of genes involved in petal and stamen symmetry development across reproductive wild type *Antirrhinum majus* tissues in the JI-7 background. Error bars are standard deviations of samples. ND: expression not determinable; DAA: days after anthesis; *p* values from *T* tests performed on the bracketed tissues. Note: stage-14 corresponds to flower anthesis

We found that upstream regulators of dorsal flower identity, *AmCYC* and *AmDICH*, have relatively high expression in tissues with petals and stamens—inflorescences and entire flower buds (Fig. 3c, d). This is consistent with their singular role in establishing dorsal petal and stamen identity [20, 21]. We found *AmCYC* and *AmDICH* expression to be sparingly low to undetectable in isolated carpel tissue of any stage (Fig. 3c, d).

**Fig. 4:**
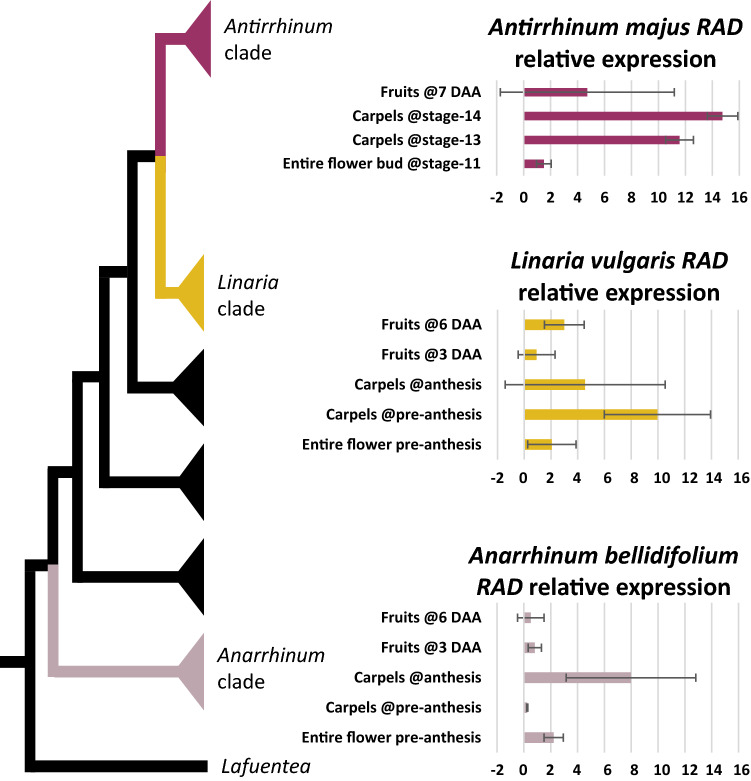
Relative expression of *RAD* orthologs in three representative species in the tribe Antirrhineae. The phylogenetic tree represents relationships among these species within the tribe [simplified from 103]

We found that the dorsal flower identity gene *AmRAD*, is expressed in tissues with petals and stamens—inflorescences and entire flower buds (Fig. 3a), consistent with its previously identified role in establishing dorsal petal and stamen identity [24]. In addition, we found a striking pattern, whereby *AmRAD* expression peaks in late stages of carpel development, in stage-14 (anthesis) flowers (Fig. 3a). We sequenced the qRT-PCR amplicon from stage-14 carpels and confirmed that the primers were amplifying the correct template. The late high expression of *RAD* is apparently conserved in the tribe Antirrhineae. The *AmRAD* orthologs in an early diverging member (*Anarrhinum bellidifolium*, *AbRAD*) and a late diverging member (*Linaria vulgaris*, *LvRAD*) have a peak of expression in carpels at anthesis (Fig. 4). The gene *AmRADlike9* has been recently reported from the *A. majus* genome sequence [47]. We report that *AmRADlike9* is sister to *AmRAD*; the duplication pre-dates the diversification of Antirrhineae (Additional file 1: Fig. S1). Unlike its paralog, *AmRADlike9* has no, or low, expression in carpel tissues (Fig. 3b), but has high expression in vegetative tissue.

**Fig. 5:**
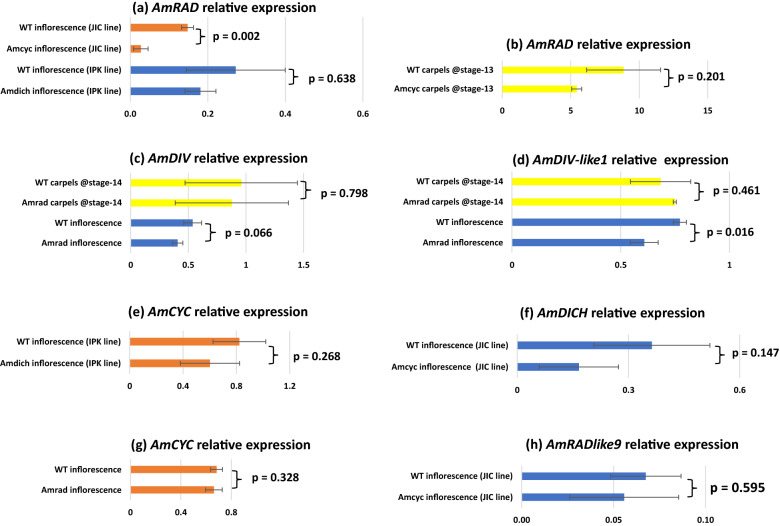
Relative expression of genes involved in petal and stamen symmetry development in mutant lines of *A. majus.* **a** *AmCYC* controls *AmRAD* transcription in inflorescences demonstrating that AmCYC–*AmRAD* interaction can be tested by qRT-PCR. Effect of *Am*dich mutation on *AmRAD* transcription is not testable by this method. **b** *Am*CYC does not control *AmRAD* transcription in carpels. **c** *AmDIV* expression in inflorescences or stage-14 carpels is not under the control of *AmRAD*. **d** *AmDIV-like1* expression under the control of *AmRAD* in inflorescences but not in carpels. **e** *AmCYC* expression is not altered in inflorescences of *Am*dich single mutant; note that from (**a**), downstream effects of *Am*dich single mutants are difficult to quantify. **f** *AmDICH* is not under the transcriptional control of *Am*CYC in inflorescences. **g** *AmCYC* is not under the transcriptional control of *Am*RAD in inflorescences. **h** *AmRADlike9* is not under the transcriptional control of *Am*CYC in inflorescences. Error bars are standard deviations of samples; *p* values from *T* tests performed on the bracketed tissues. Note: stage-14 corresponds to anthesis; *Am*dich mutant is from a different background than other mutants. Error bars are standard deviations of samples. DAA: days after anthesis

Similar to *AmRAD*, the other *MYB* genes associated with floral symmetry—*AmDRIF1*, *AmDRIF2*, *AmDIV*, and also *AmDIV-like1*—are expressed in carpel tissue but are not localized in the dorsal or the ventral locule (Fig. 3e–h). However, a pattern of localization emerges between two tissues: carpel wall (plus style) vs. ovules (plus septum and placenta). *AmRAD* is upregulated in the carpel wall relative to the ovules (Fig. 3a), whereas *AmDIV-like1* has the opposite localization, being upregulated in in the ovules (Fig. 3f). This provides evidence that a possible competitive interaction between *AmRAD* and *AmDIV-like1* may define the development of the two distinct regions of a carpel—the wall and the fertile tissue within. The pattern of localization of *AmDIV-like1* that we detect through qPCR (Fig. 3f) is consistent with the in situ mRNA hybridization assays done by previous workers—such as says detect a higher expression of *AmDIV-like1* in ovules than in the carpel wall [26]. We did not have access to Amdiv-like1 mutants [26], but we tested for *AmDIV-like1* expression in Amrad mutant background (Fig. 5d, next section).

### Transcriptional regulatory interactions are limited to positive regulation of *AmRAD *by *Am*CYC

We determined levels of *A. majus* flower symmetry gene expression in available *Amcyc*, *Amdich* and *Amrad* genetic backgrounds (seed sources in Table 1). These data confirm positive regulation of *AmRAD* by *Am*CYC in the inflorescences (Fig. 5a) [24] suggesting that qRT-PCR is an appropriate tool to test for such interactions. Beyond the *Am*CYC–*AmRAD* regulatory interaction, we found evidence for only one other transcriptional regulatory interaction: *AmDIV-like1* expression was significantly reduced in *Amrad* inflorescences compared to the wildtype (Fig. 5d). The pattern was in the same direction, but not significant, for *AmDIV* expression in *Amrad* inflorescences compared to WT (Fig. 5c). Interestingly, the same pattern of reduced *AmDIV/AmDIV-like1* expression in the *Amrad* background was not seen in carpel tissues (Fig. 5c, d). *AmCYC* does not control the transcription of *AmRADlike9*, the sister gene of *AmRAD* (Fig. 5h). *AmRADlike9* has one predicted TCP-binding site within the first 3000 bp upstream of its translational start site (Additional file 5: Table S5), suggesting that one such site is insufficient for activation by *Am*CYC homologs. We had earlier predicted a cross-regulation between *AmCYC* and *AmDICH* based on predicted TCP-binding sites [43] but qRT-PCR data provides no such evidence (Fig. 5e, f).

**Table 1:** Seed sources

Line	Wildtype ID	Mutant ID	Source
*AmCYC*	JI-7	JI-608	The John Innes Centre (JIC), UK
*AmDICH*	MAM-428	MAM-95	The Leibniz Institute of Plant Genetics and Crop Plant Research (IPK), Germany
*AmRAD*	JI-7	JI-654	The John Innes Centre (JIC), UK
*AmDIV*	JI-7	JI-13	The John Innes Centre (JIC), UK
*Solanum lycopersicum*	Microtom	Not applicable	Provided by Dr. Vivian Irish, Yale School of Medicine, USA
*Linaria vulgaris*	Accession 15127	Not applicable	B&T World Seed
*Anarrhinum bellidifolium*	Accession 1682	Not applicable	University of Copenhagen Botanical Garden, Denmark

### Expression of *SlTCP7*, *SlTCP26*, *SlRADlike4*, *SlDIVlike5*, and *SlDIVlike6* suggests potential interaction

**Fig. 6:**
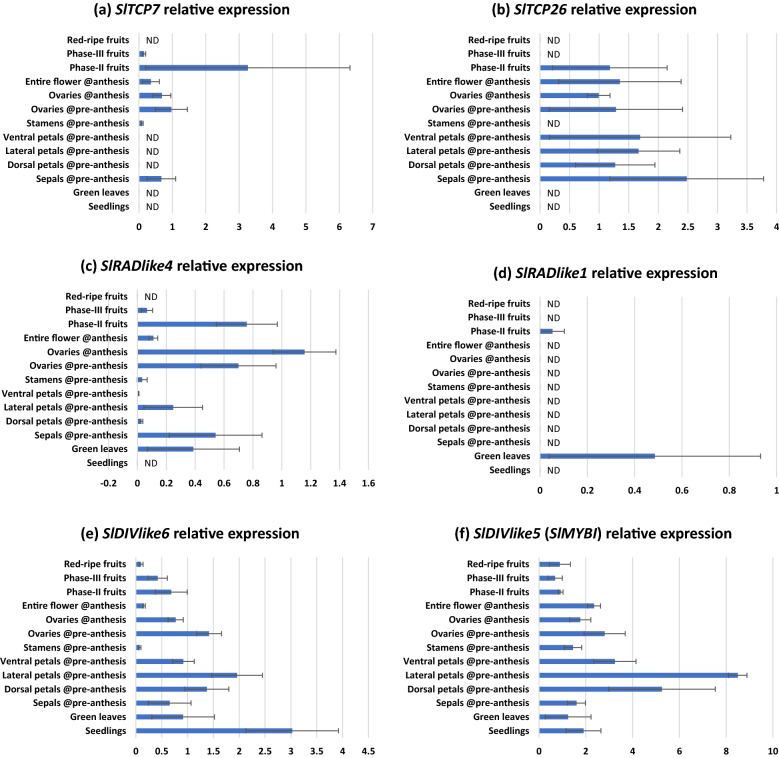
Relative expression of *CYC*, *RAD* and *DIV* orthologs and one *DIV* paralog in wildtype *Solanum lycopersicum* across reproductive organs. Error bars are standard deviations of samples. ND: expression not determinable. Note: anthesis is stage-20; pre-anthesis is stage-16

We used qRT-PCR to determine relative expression of the homologs of *A. majus* flower symmetry genes in *S. lycopersicum* (Table 2). We found that all the *S. lycopersicum* genes tested, except for *SlRADlike1*, are broadly expressed across tomato vegetative and reproductive tissues (Fig. 6). Overlapping expression is an important criterion for genes/gene products to interact with each other. Interestingly, the expression of these genes overlaps in carpels and fruits, and is often high in those tissues. This suggests that these genes may have a key role in carpel and fruit development. This is consistent with the previously described interaction of SlRADlike4 and SlDIVlike5 in tomato fruits, where these two proteins compete for SlFSB1 [41], which is a paralog of *Am*DRIF1&2 [27]. In addition, the expression of *SlTCP7* and *SlTCP26* (orthologs of *AmCYC*/*AmDICH*) is not dorsally restricted in flowers (Fig. 6). Instead, *SlTCP26* has a pan-floral expression, and both *SlTCP7* and *SlTCP26* are strongly expressed in the developing fruit as previously demonstrated [19, Tomato Expression Atlas by 44].

**Table 2:** Orthologs of genes associated with a CYC–RAD–DIV–DRIF interaction

Species	AmCYC	AmRAD	AmDIV	SlDIVlike5 (SlMYBI, Tomato fruit, see note)	DRIF Group2a (AmDRIF1)	DRIF Group2b (AmDRIF2)	DRIF Group1 (SlFSB1, tomato fruit)
*Antirrhinum majus*	AmCYC, AmDICH	AmRAD, AmRADlike9	AmDIV, AmDIV-like1	AmDIVlike10, AmDIVlike13, AmDIVlike11	AmDRIF1, AmDRIFlike6, AmDRIFlike7	AmDRIF2, AmDRIFlike3	AmDRIFlike4, AmDRIFlike5
*Solanum lycopersicum*	SlTCP7, SlTCP26	SlRADlike4 (FSM1), SlRADlike1	SlDIVlike6	SlDIVlike5 (MYBI), SlDIVlike10	SlDRIF1, SlDRIF3	SlDRIF2	SlDRIF5 (FSB1), SlDRIF4
*Vitis vinifera*	GSVIVT01036449001	GSVIVT01031975001	DIVlike8	DIVlike12, DIVlike7	DRIFlike2	DRIFlike1, DRIFlike3	DRIFlike4
*Oryza sativa*	Os03g49880 (OsTB1)	Os05g50350, Os12g33950, Os02g47744	DIV-like3, DIV-like4, DIV-like5	DIV-like1, DIV-like2	DRIFlike1, DRIFlike2	DRIFlike1, DRIFlike2	DRIFlike3, DRIFlike4, DRIFlike5, DRIFlike6
References	[16, 102]	Additional file 1: Fig. S1, [45, 63]	Additional file 1: Figs. S1 and S4, [45, 63]	Additional file 1: Figs. S1 and S4	Additional file 1: Fig. S3	Additional file 1: Fig. S3	Additional file 1: Fig. S3

Relationships among close homologs of *SlDIVlike5* are not well-resolved beyond Solanales + Lamiales. The *V. vinifera* and *O. sativa* genes could be orthologs or close paralogs of *SlDIVlike5*

### A CYC–*RAD* regulatory interaction is present in tomato

There are two *AmCYC*/*AmDICH* orthologs in *S. lycopersicum*—*SlTCP7* and *SlTCP26*; and there are two *AmRAD/AmRADlike9* orthologs in *S. lycopersicum*—*SlRADlike1* and *SlRADlike4*. We selected *SlTCP26* and *SlRADlike4* to test for a *CYC–RAD* interaction in flowers*.* We did not select *SlTCP7,* because its expression is low in whole stage-20 flowers at anthesis relative to other tissues (Fig. 6a) making downregulation difficult to assess in VIGS experiments (data not shown). We did not select *SlRADlike1* for the following two reasons. First, *SlRADlike1* is not at all expressed in reproductive tissue, except at a low level in phase-II fruits, making it impossible to test for a CYC**–***SlRADlike1* regulatory interaction in flowers (*SlRADlike1* is expressed at a low level in phase-II fruits; however, these fruits are too small for RNA extraction, and fruits cannot be pooled for RNA extractions given the mosaic nature of VIGS). Second, *SlRADlike1* has only one predicted TCP-binding site in the upstream region (Additional file 5: Table S5), hence is unlikely to be under the control of *CYC* genes (because the only predicted TCP-binding site upstream of *AmRADlike9* could not evoke upregulation by *Am*CYC, Fig. 5h).

We suspected that *Sl*TCP26 transcriptionally regulates *SlRADlike4* based on the following two lines of evidence. First, these two genes are often expressed in the same tissues (Fig. 6b, c). Second, *SlRADlike4* has five predicted TCP-binding sites within the first 3000 bp upstream of its translational start site (Additional file 5: Table S5). We have previously demonstrated that *RAD* genes that are known or predicted to be under the transcriptional control of CYC proteins are significantly enriched in predicted TCP-binding sites in the first 3000 kb upstream of their translational start sites [43].

**Fig. 7:**
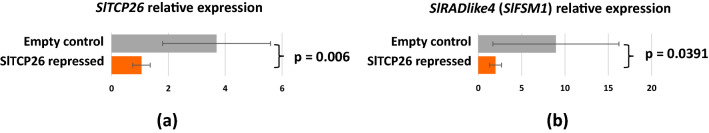
Downregulation of *SlTCP26* (**a**) and its effect on *SlRADlike4* (**b**)*.* Error bars are standard deviations of samples. The *p* values are from *T* tests performed on the bracketed tissues assuming equal variances (determined by Levene’s Test). Samples sizes are eight and six, respectively, for control and repressed lines

We downregulated *SlTCP26* expression in tomato employing VIGS (Fig. 7a) and confirmed downregulation in stage-20 (anthesis) flowers. We found a concomitant decline in *SlRADlike4* expression in the same tissues (Fig. 7b). This provides strong evidence that *SlRADlike4* is positively regulated by *Sl*TCP26*.* We predict this transcriptional control to be direct—*Sl*TCP26 likely binds to the predicted TCP-binding sites present upstream the translational start site of *SlRADlike4* (Additional file 5: Table S5). This provides preliminary evidence of a CYC–*RAD* regulatory interaction in tomato.

### A CYC–*RAD* regulatory interaction is likely ancestral to Lamiales + Solanales

**Fig. 8:**
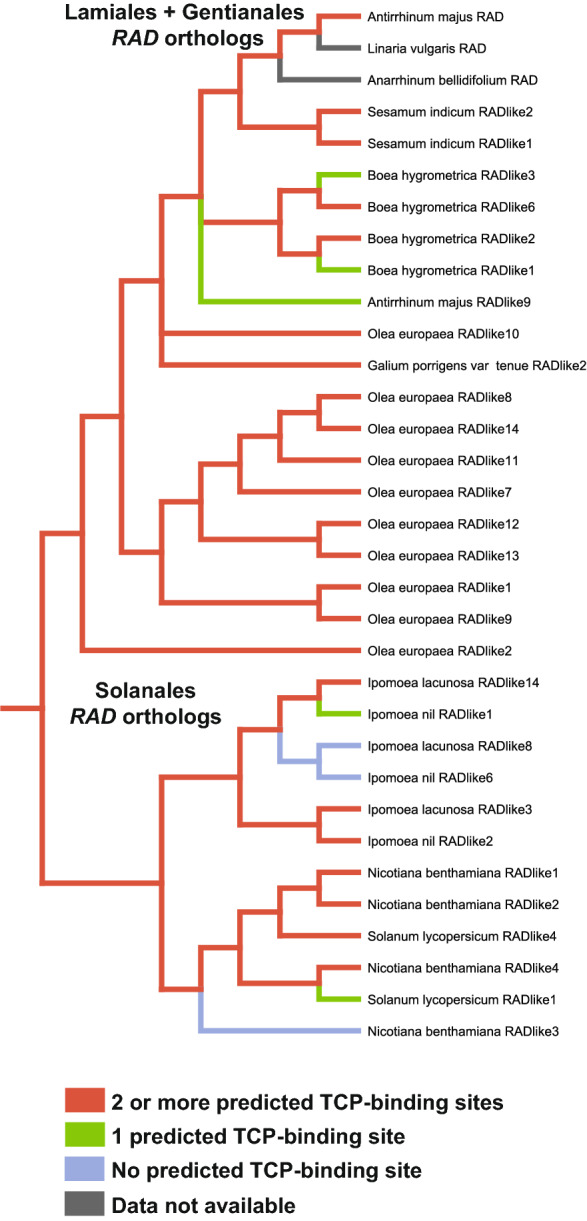
Parsimony based ancestral state reconstruction of the number of predicted TCP-binding sites present within the first 3000 bp upstream of the translational start sites of *AmRAD* orthologs in Lamiales and Solanales. The ancestral *RAD* gene had at least two predicted TCP-binding sites upstream of its translational start site

We predicted TCP-binding sites within the first 3000 bp upstream of the translational start sites of *AmRAD* orthologs in Solanales and Lamiales (Additional file 5: Table S5), then estimated the ancestral state of this character across Lamiales + Solanales. Presence of at least two predicted TCP-binding sites in the 3000 bp upstream region is homologous between *AmRAD* and *SlRADlike4*, and is ancestral to Lamiales + Solanales (Fig. 8). This provides predictive, bioinformatic evidence that the CYC–*RAD* interaction seen in *A. majus* and *S. lycopersicum* are homologous.

## Discussion

### Expression of *AmRAD*, *AmDIV*/*DIV*-*like1*, and *AmDRIF1&2* are consistent with a function in carpel development independent of dorso-ventral identity

We identified a novel peak in *AmRAD* expression late in carpel/fruit development. This indicates a potentially important developmental function in later stages of carpel/fruit development, especially in the carpel wall, where *AmRAD* expression is highest. This function is likely independent of fruit symmetry, because the key genes associated with corolla symmetry—*AmCYC*, *AmDICH*, *AmRAD*, *AmDIV*, as well as *AmDIVlike1*—are either expressed at statistically equivalent levels in both the dorsal and the ventral locules, or are not significantly expressed in carpels at all (Fig. 3). *AmCYC* is expressed at extremely low levels in carpels (Fig. 3c) but this is likely background expression and not functional because of the following two reasons. First, because *AmRAD* expression remains unaltered stage-13 carpels of *Amcyc* mutants (Fig. 5b). Second, because later in fruit development, (fruits 11 days after anthesis), *AmRAD* continues to express even though *AmCYC* (and *AmDICH*) are not expressed (Fig. 3a, c, d). *CYC* orthologs in early diverging Lamiales are expressed in the carpels [36]. It is possible the expression in carpels has been lost/reduced in the line leading to *AmDICH* and *AmCYC.*

*Am*CYC–*AmRAD* interaction has a non-cell-autonomous function in defining the monosymmetric carpels/fruits in *A. majus,* where the two locules of the carpels/fruits have distinct morphology (images of mutant in Additional file 1: Fig. S2) [unpublished data available in thesis 48]. This function does not involve dorsoventrally distinct transcription of *AmCYC* or *AmRAD* in the carpels, but likely involves the movement of AmRAD proteins from the dorsal petals to the dorsal locule of the carpel [similar to migration to lateral petals hypothesized in 24]. This mutant morphology is consistent with that seen in the fruits of the cyc mutant of another Lamiales species *Misopates orontium* [49]. However, carpel monosymmetry is patterned in early stages of carpel development, and not at or near flower anthesis (where the peak of *AmRAD* transcription is seen, Fig. 3a), and this function in carpel symmetry is dependent on *AmCYC*. Hence, we predict that *AmRAD* has two separate functions in carpel/fruit development. First, an *AmCYC*-dependent, non-cell-autonomous function in patterning carpel symmetry during the early stages of development. Second, an *AmCYC*-independent, cell-autonomous function in later stages of development. In our initial surveys, we did not find any morphological differences between the WT and Amrad fruits that could be linked to the second function (and not the function in fruit symmetry). Determining the phenotype of the second function would require extensive anatomical analyses best suited for a follow-up study.

The peak of *AmRAD* transcription in carpels at anthesis (Fig. 3a) likely controls the second, hitherto untested phenotype in the later stages of carpel/fruit development. It is likely that this function involves *Am*RAD competitively excluding *Am*DIV/DIV-like1 from interacting with *Am*DRIF1/2. This hypothesis is based on the following lines of evidence. First, high expression of *AmRAD* in carpels coincides with expression of *AmDIV/DIV-like1* and *AmDRIF1&2* in those tissues, and second, the only biochemical interactions known for *Am*RAD homologues involve competition with *Am*DIV/DIV-like1 homologs for *Am*DRIF1/2 interaction. *Am*CYC/*Am*DICH downregulate *AmDIV* in stage-10 flowers [26] possibly by upregulating *Am*RAD which in turn may disrupt *AmDIV* autoregulation. However, we find that the *Amrad* mutant background does not alter *AmDIV* expression in stage-14 carpels or in inflorescences (Fig. 5c).

Hence, the *AmCYC/AmDICH* control over *AmDIV* is either limited to stage-10 flowers or is mediated by factors other than *Am*RAD. We had predicted an *Am*CYC–*Am*DICH cross-regulation [43], but do not find any evidence for *Am*CYC transcriptionally regulating *AmDICH* (Fig. 5f). The effects of *Am*dich mutation on downstream genes is difficult to quantify in single mutants (Fig. 5a) [24], but we predict them to be similar to *Am*cyc. Therefore, it is unlikely, that *Am*CYC–*Am*DICH regulate each other, or even themselves. The predicted TCP-binding sites upstream of *AmCYC* and *AmDICH* are potentially bound by other TCP proteins, as in *Gerbera hybrida* [50]*.* Alternatively, *Am*CYC/*Am*DICH have a complex interaction—this is based on the evidence that in *Torenia fournieri*, another Lamiales species, the expression of a *CYC* ortholog *TfCYC1* declines irrespective of whether another ortholog *TfCYC2* is upregulated or downregulated [33]. We also report that *AmRAD* does not affect the transcription of *AmCYC*, unlike its ortholog *TfRAD1* in *Torenia fournieri* [33].

### A conserved ancestral function of RAD–DIV–DRIF in fruits may pre-date Lamiales flower monosymmetry

In Lamiales, *Am*RAD is known to function in defining floral monosymmetry along the dorso-ventral axis, and monosymmetry evolved in Lamiales after its from its close relative Solanales. *Solanum lycopersicum* is a model species in the order Solanales, and in whose fruits an RAD–DIV–DRIF like interaction has been reported [41]. In this interaction, the RAD component suppresses cell expansion in the pericarp tissue. Pericarp, or the fruit wall, is the ovary wall after fertilization. We provide suggestive evidence that *Am*RAD has a function in late carpel/fruit development, and that this function may involve *AmDIV*, *AmDIV-like1*, and *AmDRIF1*&*2* in that expression of these gene overlaps with expression of *AmRAD* in later stages of carpel development. Hence, we hypothesize an ancestral function of *RAD*-like genes is in controlling micromorphology during carpel wall development. An *RAD* function in carpels is likely ancestral to Lamiales—*RAD* is expressed in the carpels of early diverging Lamiales [36], as well as in later diverging Lamiales—Plantaginaceae (this study), Phrymaceae [51], and Lamiaceae [51]. Similarly *CYC* is expressed in the carpels of early diverging Lamiales [36], Phrymaceae, [51], and Lamiaceae [51], with an exception in *A. majus* (where expression is low or undetectable). This suggests that a *CYC* and *RAD* co-expression, and possibly, interaction, in carpels is ancestral to Lamiales, with a later loss of *CYC* expression in Antirrhineae carpels. This also suggests that the RAD–DIV–DRIF interaction, which is crucial in defining Lamiales monosymmetry, did not evolve during the origin of flower monosymmetry in Lamiales but was co-opted from a different function, likely fruit/carpel development, to define the dorso-ventral monosymmetry in Lamiales flowers.

### *SlTCP26* transcriptionally regulates *SlRADlike4* in tomato

Downregulating *SlTCP26* by VIGS leads to a corresponding decrease in *SlRADlike4* expression. This provides strong evidence for transcriptional control of *SlRADlike4* by *Sl*TCP26. However, our data do not provide evidence as to whether this interaction is direct (*Sl*TCP26 protein binding to the 5′ *cis*-regulatory sequence of *SlRADlike4*) or indirect (downstream targets of *SlTCP26* binding to the 5′ *cis*-regulatory sequence of *SlRADlike4*). TCP proteins (similar to *Sl*TCP26) are known or predicted to be transcription factors that bind to the consensus TCP-binding site 5′–GGNCCC-3′ [35, 52, 53]. *RAD* orthologs that are known or predicted to be under the direct transcriptional regulation by *CYC* orthologs are likely to be enriched in predicted TCP-binding sites in the first 3000 kb upstream their translational start site [43]. *SlRADlike4* has five such predicted TCP-binding sites within the first 3000 kb upstream of its translational start site. Together, the data from bioinformatics analysis and gene silencing experiments suggest that *Sl*TCP26 directly upregulates the transcription of *SlRADlike4*. Whether the transcriptional control of *SlRADlike4* by *SlTCP26* is direct can be verified by DNA–protein interaction studies. One such test could be a yeast-hybrid assay that determines whether the protein *Sl*TCP26 can activate transcription by acting on wild-type promoter of *SlRADlike4* but cannot activate transcription when the GGNCCC sites in the promoter are modified or deleted. Such studies are beyond the scope of this work. There were no noticeable differences between the populations treated with empty pTRV2 vs. pTRV2-*SlTCP26* in terms of flower size and symmetry, and petal number (data not shown). However, it is possible that *SlTCP26* controls micromorphological features, like cell number or size, in flowers. Tomato plants often bear flowers with additional floral organs in any whorl (called ‘megablooms’ in horticulture). Such megabloom flowers appeared in untreated wildtype, empty pTRV2 treated, and pTRV2-*SlTCP26* treated populations. Therefore, it is unlikely that VIGS-associated downregulation of *SlTCP26* is responsible for this phenotype. The population treated with pTRV2-*SlTCP26* developed flower buds *ca*. 10 days before the empty pTRV2 treated population. Further experiments are needed to quantify this shift. It is not surprising that silencing of a *CYC* ortholog did not have obvious morphological effects in *S. lycopersicum*, even though molecular testing confirms a downregulation. Tracking the function of the *CYC* ortholog *AtTCP1* in *Arabidopsis thaliana* has also been difficult. Traditional silencing methods (including RNA interference) could not reveal the function of *AtTCP1* [54, 55]. The function of *AtTCP1* was revealed when a chimeric *AtTCP1* fused to a transcriptional repressor domain was over-expressed [55]. However, this method is not appropriate for studying the function of *SlRADlike4* or its upstream regulator *SlTCP26,* because strong downregulation of *SlRADlike4* kills all transformants [41].

### CYC–RAD–DIV–DRIF interaction was likely co-opted to flower monosymmetry from other functions

A CYC–RAD–DIV–DRIF interaction defines flower monosymmetry in Lamiales. A part of this interaction, RAD–DIV–DRIF interaction, is present in Solanales, and affects fruit development in tomato [41]. We provide preliminary evidence that the RAD–DIV–DRIF interaction is conserved across Lamiales + Solanales carpel/fruit development. Here we report a CYC–*RAD* interaction in tomato, where *Sl*TCP26 transcriptionally upregulates *SlRADlike4* (Fig. 7b). This would suggest that the entire CYC–RAD–DIV–DRIF interaction is likely ancestral to Lamiales + Solanales, and was co-opted *en bloc* to define the novel phenotype of flower monosymmetry in Lamiales. However, this conclusion is diminished by the fact that *AmRAD* and *SlRADlike4* have sister genes that we demonstrate or predict to not be under the control of *Am*CYC and *Sl*TCP26. These two contrasting lines of evidence can be explained by two hypotheses. First, the CYC–*RAD* interaction in *A. majus* and *S. lycopersicum* are not homologous, and evolved independently. Second, the CYC–*RAD* interaction in *A. majus* and *S. lycopersicum* are homologous, but the CYC–*RAD* interaction has been lost in some paralogs. If the second hypothesis is true, then the presence of two or more predicted TCP-binding sites upstream of *AmRAD* and *SlRADlike4* should be homologous, the state being ancestral to Lamiales + Solanales.

We conservatively expect that the presence of a single TCP-binding site within the 3000 bp immediately upstream of the translational start site is insufficient to invoke regulation by AmCYC and its orthologs but having two can be sufficient. This prediction is based on the following two lines of evidence. First, we demonstrate that *AmRADlike9*, which has one predicted TCP-binding site within the first 3000 bp upstream of its translational start site (Additional file 5: Table S5), is not under the transcriptional control of *AmCYC* (Fig. 5h). Second, having two or more such sites is likely functional, because *AmRAD* [under the control of AmCYC, 52] and *AtDWARF4* [under the control of AmCYC ortholog in Arabidopsis, AtTCP1, 56] each have two such sites in their upstream region.

Our ancestral state reconstruction supports the second hypothesis that the presence of two or more predicted TCP-binding sites upstream of *AmRAD* and *SlRADlike4* is homologous, the state being ancestral to Lamiales + Solanales (Fig. 8). This provides evidence that a CYC–*RAD* interaction is ancestral to Solanales + Lamiales, with the likely ancestral function of this interaction in carpel/fruit development. The lack of significant *AmCYC* expression in *A. majus* carpels/fruit likely represents a loss, because in early diverging Lamiales, both *CYC* and *RAD* genes are expressed during carpel development [36, 51].

### Explaining the repeated recruitment of CYC–RAD–DIV–DRIF interaction

Since the initial discovery of CYC function in *A. majus* flower symmetry, *CYC* orthologs have been implicated in defining independently derived floral monosymmetry in many major clades of flowering plants [reviewed in 57]. A role for CYC–RAD–DIV interaction (DRIF participation not tested) has been suggested in the development of monosymmetric flowers in the order Dipsacales [58–61], and potentially in magnoliids [62, 63]. A similar, TCP–RAD–DIV–DRIF interaction is possibly involved in orchid monosymmetry [64, 65]. The repeated parallel recruitment of *CYC* orthologs in defining floral monosymmetry has been explained with the following model. An ancestral dorsal-specific expression of CYC was already present in the polysymmetric ancestral flowers [66]; this ancestral dorsal-specific expression would generate a bias, where *CYC* would be more likely to be co-opted in defining any new morphology evolving in the dorsal floral organs. This model is based on the observation that in *Arabidopsis thaliana*, which has non-monosymmetric flowers at maturity, the *CYC* ortholog *AtTCP1* is expressed in the dorsal region of the floral primordium [66]. The applicability of this model across eudicots has been questioned [43], because several lines of evidence demonstrate that a dorsal-specific expression is likely not ancestral to eudicots, or even Brassicaeae. Within Brassicaceae, monosymmetric flowers do not have an *Arabidopsis*-like dorsally restricted *CYC* expression in their primordia (but the expression is dorsally restricted later during petal development) [67]. *AtTCP1* expression is not limited to the dorsal side of the floral meristem, but is widely expressed in other parts of the plant [55], and this is distinct from the expression pattern of *AmCYC*/*AmDICH*. Even within Lamiales, dorsally restricted *CYC* and *RAD* expression is clearly a derived state—early diverging Lamiales (that have non-monosymmetric flowers) have pan-floral expression [36]. This expression pattern of early diverging Lamiales is consistent with the expression pattern we report in in *S. lycopersicum*. That is, the expression of *CYC* orthologs in *S. lycopersicum* is not restricted to the dorsal petals in the polysymmetric flowers of tomato, at least in later stages of flower development (this is in contrast with *A. majus*, where *AmCYC* expression remains restricted to dorsal organs even in late stages of development, namely, stage 9) [in situ in 20, PCR in 21, stage identified from 68]. This provides evidence that the dorsal-specific expression of *AmCYC*/*AmDICH* and their orthologs in later-diverging Lamiales is an innovation of Lamiales, and that the polysymmetric flowers of the ancestors of Lamiales + Solanales likely did not have such dorsally restricted *CYC* expression.

These lines of evidence demonstrate that the ancestral expression of *CYC* in Brassicales (which includes *A. thaliana*) or Lamiales + Solanales (which includes *A. majus*) was not dorsally restricted. But then, what expression pattern of *CYC* (and *RAD*, *DIV*, *and DRIF*) genes is likely ancestral across eudicots, and possibly angiosperms? There are two alternative scenarios. First, the expression pattern seen in *S. lycopersicum* (*CYC* expression pan-floral, pan-plant) is ancestral, and second, the one represented by *A. thaliana* and *A. majus* (*CYC* restricted to dorsal side of flower/floral primordium) is ancestral. There has been no evidence outside *A. thaliana,* where a flower with ancestrally polysymmetric flowers displayed a dorsally restricted *CYC* expression, but several lines of evidence support the first scenario. For example, in an early diverging eudicot, *Eschscholzia californica* (which has non-monosymmetric flowers), the expression of one of the two *AmCYC* orthologs is at organ boundaries, and the other is across the floral meristem [69], and neither is dorsally restricted. In addition, we looked in the published expression data of two other angiosperms outside the Lamiales + Solanales clade and the Brassicales clade: the rosid *Vitis vinifera* (Additional file 10) and the monocot *Oryza sativa* (Additional file 11). In these two outgroup species, we investigated the expression of the orthologs of the genes involved in *A. majus* flower symmetry (*AmCYC*, *AmRAD*, *AmDIV*, and *AmDRIF1/2*) and of their homologs involved in *S. lycopersicum* fruit development (*SlRADlike4*, *SlMYBI,* and *SlFSB1*). These orthologs in the outgroups show a plant-wide expression and are often upregulated in carpel/fruit tissue. For example, the *AmRAD* ortholog *Vitis vinifera* (GSVIVT01031975001) is strongly upregulated in carpel tissues (Additional file 10: Fig. S2), similar to the *AmDIV* ortholog *Vitis vinifera DIVlike8* that is strongly upregulated in carpels and fruits (Additional file 10: Fig. S3). Clearly, the expression of the *CYC* orthologs in Brassicales (except *A. thaliana*), in early Lamiales, in Solanales, in *V. vinifera* and in *O. sativa*, is not restricted to flowers (unlike in *A. majus*), and/or when expressed in flowers/floral meristems, the expression is not dorsally restricted (unlike in *A. majus* or *A. thaliana*). These lines of evidence support our hypothesis that the ancestral expression of *CYC* and *RAD* was not restricted to the dorsal side of flowers, and the expression pattern in *A. majus* is derived.

Hence, the model explaining the repeated recruitment of *CYC* towards defining floral monosymmetry by hypothesizing an ancestrally dorsally restricted *CYC* expression is unlikely to be applicable. Then, the question persists—why would a CYC–RAD–DIV–DRIF interaction, and not any other genetic interaction, be recruited for flower monosymmetry in Lamiales (and in other flowering plant lineages)? We provide evidence that CYC–RAD–DIV–DRIF interaction likely pre-dates the origin of flower monosymmetry in Lamiales, and its ancestral function was likely in carpel/fruit development. There is suggestive evidence that an RAD–DIV–DRIF interaction, and possibly, CYC–RAD–DIV–DRIF is ancestral to all flowering plants (or at least to magnoliids, monocots, and eudicots) and was possibly involved in carpel development, because it has been reported or hypothesized across many angiosperm lineages. For example, an RAD–DIV–DRIF interaction has been biochemically tested (but not functionally validated) in *Arabidopsis thaliana,* where at least one *RAD* ortholog is expressed in carpels (AtRL2, At2g21650) and all of the DIV orthologs can bind to a DRIF paralog (AtFSB1, At1g10820; not all DRIF homologs tested) [DRIF homology from 27, protein interaction from 41, DIV homology from 59, 70]. Expression of *CYC*, *RAD*, and *DIV* genes in carpels and fruits is a recurrent pattern in angiosperms, including in magnoliids [*CYC* in 62, *RAD* and *DIV* in 63], orchids [*RAD* and *DIV* in 71], possibly in early core eudicots [*CYC* in 69, carpels and stamens pooled as one tissue], in lamiids [*CYC* and *RAD* in 36, 51], rosids [*RAD* in 70], and campanulids [*CYC* in 50]. The evidence for this function to be in carpel development is the strongest, but is not limited to those organs. Indeed, in tomato, *CYC*, *RAD*, and *DIV* are expressed, to a varying degree, in all floral organs in addition to vegetative organs.

We propose that CYC–RAD–DIV–DRIF interaction was co-opted towards defining floral monosymmetry for the following three reasons. First, because the interaction was already available; second, because the core interaction is based on protein–protein competition from which the competing components (RAD and DIV) could be recruited to define opposite sides of the flower; and third, because co-option of CYC–RAD–DIV–DRIF interaction to flower monosymmetry would require only one evolutionary step of making *CYC* expression dorsal-specific. *CYC*, *RAD*, and *DIV* likely had a pan-floral expression in the common ancestors of Lamiales + Solanales as estimated from the expression pattern in representative species [*DIV* from this work and 27, *DRIF* from 27, *CYC* and *RAD* from this work and 36]. The ancestral expression pattern of DRIF is not clear, but given its polysymmetric expression in *A. majus* flowers, it is likely that it too was ancestrally pan-floral in expression irrespective of symmetry. This non-localized, pan-floral activity of this interaction could be partitioned to define floral monosymmetry—one side defined by a strong RAD activity, the other by a strong DIV activity, with lateral organs being defined at the boundaries of these two zones in a density-dependent manner. The strong, dorsally restricted activity of RAD can be acquired by a change in the expression pattern of its transcriptional upregulator *CYC*. The expression pattern of the other two genes, *DIV* and *DRIF*, need not have undergone any major changes. Thus, the evolution of CYC–RAD–DIV–DRIF interaction seen in monosymmetric flowers of *A. majus* from a pan-floral CYC–RAD–DIV–DRIF interaction of the polysymmetric ancestral flower would require a single evolutionary change—the expression of *CYC* having evolved a dorsally restricted pattern. In *A. majus*, this change is likely represented by the putative cis-regulatory sequence located 4.2 kb upstream of *AmCYC* translational start site. When this site is disrupted by transposon insertion in the backpetal mutants, the expression of *AmCYC* becomes pan-floral [21].

Existing genes are often recruited to define novel phenotypes [72, 73]. Co-option of single genes in defining novel phenotypes has been reported from a wide variety of organisms, including the co-option of *CYC* to define flower monosymmetry [73–78]. We provide preliminary evidence that the CYC–RAD–DIV–DRIF interaction that defines flower monosymmetry in Lamiales was co-opted *en bloc* from another function, likely female organ development, and was not assembled de novo near the base of Lamiales. This is consistent with the *en bloc* co-option reported in other organisms. [72, 75, 79, 80]. Our results add to the evidence that evolution of novel phenotypes can be associated with or facilitated by the co-option of entire genetic interactions.

## Conclusions

The CYC–RAD–DIV–DRIF interaction is critical for flower symmetry in Lamiales, but its origin had remained unresolved. We provide preliminary support to the hypothesis that this program was co-opted *en bloc* from a function in carpel/fruit development. We also raise the hypothesis that the program is ancestral to a wider group of flowering plants and was hence recruited repeatedly towards defining independently derived-monosymmetric flowers. This is in line with the idea that the evolution of novel traits is facilitated by co-option of existing regulatory interactions.

## Materials and methods

### Plant material

The following species were studied in this work: *Antirrhinum majus* L., Sp. Pl. 2: 617 (1753), *Solanum lycopersicum* L., Sp. Pl. 1: 185 (1753), *Linaria vulgaris* Mill., Gard. Dict., ed. 8. [unpaged] *Linaria* no. 1 (1768), and *Anarrhinum bellidifolium* Fenzl ex Jaub. & Spach, Illustr. Pl. Or. v. 54 (names from www.ipni.org). Seed sources are listed in Table 1. We imported *A. majus* seeds under USDA-APHIS permit P37-16-01034. We germinated and maintained the plants under 16 h daytime at 20–26 °C.

### qRT-PCR tissue sampling

We collected *A. majus* tissue (Fig. 2 and Additional file 5: Table S1) whose developmental stages were determined from a published developmental series [68] or by us. We did not sample organ primordia, because gene expression is known in those stages [20, 21, 24, 26]. We collected *Linaria vulgaris* and *Anarrhinum bellidifolium* tissue from developmental stages comparable to *A. majus*. We collected *S. lycopersicum* tissues (Additional file 5: Table S2) based on published developmental series [81, 82]. Dorsal and ventral positions were determined relative to the main axis—we dissected flowers with the awareness that *S. lycopersicum* flowers are partly rotated relative to the main axis [83], the carpels are oblique relative to the median plane of the flower [84, 85], and that the inflorescences are sympodial [86]. It can be difficult to determine what developmental stages are equivalent between *S. lycopersicum* and *A. majus* for two reasons. First, because a detailed atlas of *A. majus* fruit development is not available (unlike for *S. lycopersicum,* which are prized for their fruits). In addition, second, because fruits of *A. majus* are capsules—they undergo a process of drying and death—unlike the fleshy fruits of *S. lycopersicum*. However, the peaks of expression patterns we detect are at or around anthesis. We consider these stages (at/around anthesis) to be equivalent between fruits of *S. lyc*opersicum and *A. majus*. This is also apparent morphologically. For example, neither of the fruits undergo their characteristic, rapid enlargement in these stages, and do not abscise their styles—all of which happen at later stages. Given these morphological similarities and the fact that Solanales and Lamiales are close relatives, we consider carpels at or around anthesis to be developmentally equivalent between *S. lycopersicum* and *A. majus*, and hence, justified for comparative analysis.

### Identifying homologs

Gene sources are listed in Additional file 5: Table S3. We isolated *RAD* orthologs from *L. vulgaris* and *A. bellidifolium* by PCR (Bullseye Taq DNA polymerase, Midwest Scientific, St. Louis, MO, USA) using degenerate primers [31]. We generated the following phylogenies to identify the relationship among *RAD*, *DIV*, and *DRIF* homologs. First, a tree of *RAD* and *DIV* genes in Lamiales, Solanales, and Gentianales (phylogeny in Additional file 1: Fig. S1, alignment and command block in Additional file 2, unedited coding sequences in Additional file 4). Second, a tree of *DRIF* genes in monocots and eudicots (phylogeny in Additional file 1: Fig. S3, alignment and command block in Additional file 6, unedited coding sequences in Additional file 7). Third, a tree of *DIV* genes in angiosperms (phylogeny in Additional file 1: Fig. S4, alignment and command block in Additional file 8, unedited coding sequences in Additional file 9). Homologs were translationally aligned using MAFFT [87]. Phylogeny was estimated using MrBayes 3.2.6 [88] available at CIPRES [89www.phylo.org]. The homology among *A. majus*, *S. lycopersicum, Vitis vinifera*, and *Oryza sativa* genes is listed in Table 2.

### Quantitative RT-PCR

We extracted total RNA from three biological replicates of each tissue (five biological replicates for carpel tissue in Fig. 5c) type using RNeasy plant minikit (Qiagen, Germantown, MD, USA) or TRI Reagent (Thermo Fisher Scientific, Waltham, MA, USA), followed by DNase treatment (TURBO DNase, Thermo Fisher Scientific), and cDNA synthesis (iScript cDNA Synthesis Kit, Bio-Rad, Hercules, CA, USA). We performed qRT-PCR with three technical replicates from each biological replicate in a StepOnePlusTM Real-Time PCR System (Thermo Fisher Scientific) using SYBR Select Master Mix (Thermo Fisher Scientific, for *AmCYC*, *AmDICH*, *AmRAD*, *AmDIV, AmDRIF1,* and *AmDRIF2*), Bullseye EvaGreen qPCR Mastermix (Midwest Scientific, for *AmDIV-like1*, and all *S. lycopersicum* genes), and PowerUp SYBR Green Master Mix (Thermo Fisher Scientific, for *AbRAD*, *LvRAD*, and *AmRADlike9*). We normalized expression of target genes in *A. majus* against *AmUBIQUITIN5* (*AmUBQ5*), or its homologs in *A. bellidifolium* and *L. vulgaris* [43, previously used by 90]. We sequenced the *AmRAD* qRT-PCR product for stage-14 carpels to confirm that the primers had amplified the correct gene. We normalized expression of target genes in *S. lycopersicum* against *Elongation factor 1-alpha* (*SlEF1a*) [91]. We determined primer efficiencies using DART [92] and analyzed expression employing the ΔΔCt method [93, 94]. Primers are listed in Additional file 5: Table S4.

### Virus-induced gene silencing

Knocking out *SlRADlike4* (*SlFSM1*) function is lethal [41]. We suspected that knocking out any putative transcriptional upregulator of *SlRADlike4* could similarly kill all transformants by terminating *SlRADlike4* transcription. Therefore, instead of strongly knocking out the expression of the putative upstream regulator by stable transformation, we employed VIGS that downregulates target genes partially, transiently, and in mosaics. We used the pTRV1/2 system to downregulate *SlTCP26* [95–97]. We acquired unmodified pTRV1/2 vectors from The *Arabidopsis* Resource Center (abrc.osu.edu), amplified a 416 bp fragment of the *SlTCP26* cDNA and cloned it into pTRV2 using NEBuilder HiFi DNA Assembly Master Mix (NEB). The insert encompasses coding and non-coding regions near the 3′ end of the transcript and can target both transcripts variants of *SlTCP26* (HM921069.1 and XM_010319513.2). We used *Agrobacterium tumefaciens* GV3101 to introduce the pTRV1/2 into tomato seedlings [as described in 95]. As a control, we infiltrated some plants with the empty pTRV2 vector (without the insert) along with the pTRV1. We sampled whole flowers at anthesis (stage-20) to test for downregulation (using extraction and qRT-PCR methods described above). Six pTRV2-insert flowers and eight control flowers (from different plants) were used for testing downregulation of *SlTCP26* and *SlRADlike4*. We compared the mean expression of these genes in the control and experimental sets by *T* test. In addition, we performed VIGS on *S. lycopersicum PHYTOENE DESATURASE* (*SlPDS*) in a parallel experiment to visually estimate the efficiency of downregulation (data not shown). The pTRV2-*SlPDS* construct targeted the same region of the native *SlPDS* transcript as in a previously published work [95].

### Putative CYC binding site ancestral state reconstruction

We identified the orthologs of *AmRAD* (Additional file 1: Fig. S1) and downloaded 3000 bp upstream of their translational start sites (Additional file 3). We selected species that are early-, mid-, and late-diverging within orders Lamiales and Solanales, and a species from the order Gentianales (Lamiales: *Olea europaea*, *Dorcoceras hygrometricum*, *Antirrhinum majus*, *Sesamum indicum*; Solanales: *Ipomoea nil*, *Ipomoea lacunosa*, *Nicotiana benthamiana*, *Solanum lycopersicum*; Gentianales: *Galium porrigens* var. *tenue*) [98]. *RADIALIS* genes are short, conserved, and have rapidly diversified in Lamiales + Solanales, making it difficult to finely resolve their relationships [phylogeny in 36, phylogeny in 43, phylogeny and interpretation in 51]. In the 3000 bp upstream region, we searched for the consensus TCP-binding site 5′–GGNCCC–3′ [35, 52, 53]. It was not possible to determine homology among the predicted consensus TCP-binding sites across species through alignment, because the sites are only six base pairs and the flanking regions are divergent (as expected from fast-evolving, non-coding sequence). Therefore, we estimated the ancestral state by scoring our tree of *RAD*-orthologs (Additional file 1: Fig. S1) for the number of predicted TCP-binding sites in the 3000 bp region (irrespective of location). We scored for three states: no, one, two-or-more predicted TCP-binding sites. Having two or more such sites is likely functional, because *AmRAD* [under the control of *AmCYC*, 52] and *AtDWARF4* [under the control of *AmCYC* ortholog in *Arabidopsis*, *AtTCP1*, 56] each have two such sites in their upstream region. We performed parsimony-based ancestral state reconstruction in Mesquite 3.61 [99].

### Identifying expression of *CYC*, *RAD*, *DIV*, and *DRIF* genes from expression atlas

We acquired expression data for these genes from *Vitis vinifera* and *Oryza sativa*. These two species are located outside Lamiales + Solanales, and hence can serve as outgroups. We acquired expression data from the publicly available expression maps at bar.utoronto.ca. This website incorporates material from previously published sources for *V. vinifera* [100] and *O. sativa* [101].

## Supplementary Information

**Additional file 1: Fig. S1.** Bayesian phylogeny of *RAD* and *DIV* genes from Lamiales, Solanales, and Gentianales. The tree was rooted at the mid-point. Posterior probabilities presented at nodes. Names of genes studied with quantitative PCR in larger font. **Fig. S2.** Dry fruits of *Antirrhinum majus*. **(a)**. Wildtype in lateral view. **(b)**. *Amcycloidea* in lateral view. **(c)**. Wildtype in top view. **(d)**. *Amcycloidea* in top view. Left side is dorsal in (a) and (b). Top is dorsal in (c) and (d). The dorsal locule acquires a ventral identity in the *Amcycloidea* mutant. **Fig. S3.** Bayesian phylogeny of *DRIF* genes in monocots and eudicots. Posterior probabilities presented at nodes. The tree was rooted at the mid-point. Genes with known DIV–DRIF interaction in larger font. **Fig. S4.** Bayesian phylogeny of *DIV* genes in angiosperms. Posterior probabilities presented at nodes. The tree was rooted at the mid-point.

**Additional file 2:** Alignment and command block for Bayesian phylogenetic analysis of *RAD* and *DIV* genes used in Additional file 1: Fig. S1.

**Additional file 3:** Up to 3000 bp upstream of translational start sites of *AmRAD* orthologs.

**Additional file 4:** Unedited coding sequences of *RAD* and *DIV* genes used in Additional file 1: Fig. S1.

**Additional file 5:** Table S1. *Antirrhinum majus* tissue collected for qRT-PCR. Table S2. *Solanum lycopersicum* tissue collected for qRT-PCR. Table S3. Source of genes used in this study. Table S4. PCR primers. Table S5. Predicted TCP-binding sites within the first 3000 bp immediately upstream of *AmRAD* orthologs.

**Additional file 6:** Alignment and command block for Bayesian phylogenetic analysis of *DRIF* genes used in Additional file 1: Fig. S3.

**Additional file 7:** Unedited coding sequences of *DRIF* genes used in Additional file 1 Fig. S3.

**Additional file 8:** Alignment and command block for Bayesian phylogenetic analysis of *DIV* genes in angiosperms used in Additional file 1: Fig. S4.

**Additional file 9:** Unedited coding sequences of *DIV* genes in angiosperms used in Additional file 1: Fig. S4.

**Additional file 10:** Figs. S1–S12. Expression the orthologs of *AmCYC*, *AmRAD*, *AmDIV*, *AmDRIF1/2*, *SlMYBI* (*SlDIVlike5*), and *SlFSB1* (*SlDRIF5*) in *Vitis vinifera*. Images are from bar.utoronto.ca. Some genes are represented by multiple transcripts.

**Additional file 11:** Figs. S1–S14. Expression the orthologs of *AmCYC*, *AmRAD*, *AmDIV*, *AmDRIF1/2*, *SlMYBI* (*SlDIVlike5*), and *SlFSB1* (*SlDRIF5*) in *Oryza sativa*. Images are from bar.utoronto.ca. Expression data for one of the *AmRAD* orthologs Os05g50350 was not available.

## Data Availability

The data sets supporting the conclusions of this article are included within the article (and its Additional files). *RADIALIS* gene sequences identified in this study are available in GenBank (https://www.ncbi.nlm.nih.gov/genbank/) under accession numbers MW464170 and MW464171.
