# Supplementary material for: A CYC–RAD–DIV–DRIF interaction likely pre-dates the origin of floral monosymmetry in Lamiales
Source: EvoDevo. 2022 Jan 29;13:3. doi: 10.1186/s13227-021-00187-w (PMC8801154; doi:10.1186/s13227-021-00187-w)
Supplement: Supplementary file 1 — Additional file 1: Fig. S1. Bayesian phylogeny of RAD and DIV genes from Lamiales, Solanales, and Gentianales. The tree was rooted at the mid-point. Posterior probabilities presented at nodes. Names of genes studied with quantitative PCR in larger font. Fig. S2. Dry fruits of Antirrhinum majus. (a). Wildtype in lateral view. (b). Amcycloidea in lateral view. (c). Wildtype in top view. (d). Amcycloidea in top view. Left side is dorsal in (a) and (b). Top is dorsal in (c) and (d). The dorsal locule acquires a ventral identity in the Amcycloidea mutant. Fig. S3. Bayesian phylogeny of DRIF genes in monocots and eudicots. Posterior probabilities presented at nodes. The tree was rooted at the mid-point. Genes with known DIV–DRIF interaction in larger font. Fig. S4. Bayesian phylogeny of DIV genes in angiosperms. Posterior probabilities presented at nodes. The tree was rooted at the mid-point. [file 13227_2021_187_MOESM1_ESM.zip › Additional file 1 Fig S2.pdf]

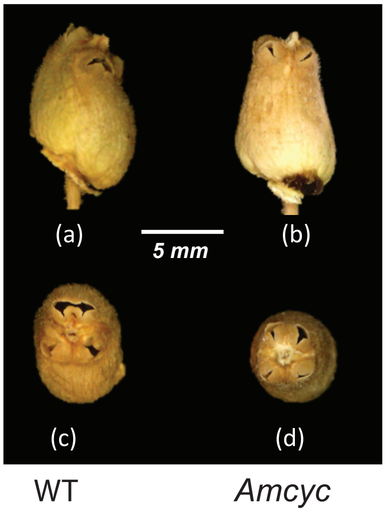

Additional file 1 Fig. S2. Dry fruits of *Antirrhinum majus*. **(a)**. Wildtype in lateral view. **(b)**. *Amcycloidea* in lateral view. **(c)**. Wildtype in top view. **(d)**. *Amcycloidea* in top view. Left side is dorsal in (a) and (b). Top is dorsal in (c) and (d). The dorsal locule acquires a ventral identity in the *Amcycloidea* mutant.
