# Supplementary material for: A CYC–RAD–DIV–DRIF interaction likely pre-dates the origin of floral monosymmetry in Lamiales
Source: EvoDevo. 2022 Jan 29;13:3. doi: 10.1186/s13227-021-00187-w (PMC8801154; doi:10.1186/s13227-021-00187-w)
Supplement: Supplementary file 10 — Additional file 10: Figs. S1–S12. Expression the orthologs of AmCYC, AmRAD, AmDIV, AmDRIF1/2, SlMYBI (SlDIVlike5), and SlFSB1 (SlDRIF5) in Vitis vinifera. Images are from bar.utoronto.ca. Some genes are represented by multiple transcripts. [file 13227_2021_187_MOESM10_ESM.pdf]

Additional file 10 Fig. S1–S12. Expression the orthologs of *AmCYC*, *AmRAD*, *AmDIV*, *AmDRIF1/2*, *SlMYBI* (*SlDIVlike5*), and *SlFSB1* (*SlDRIF5*) in *Vitis vinifera*. Images are from bar.utoronto.ca. Some genes are represented by multiple transcripts.

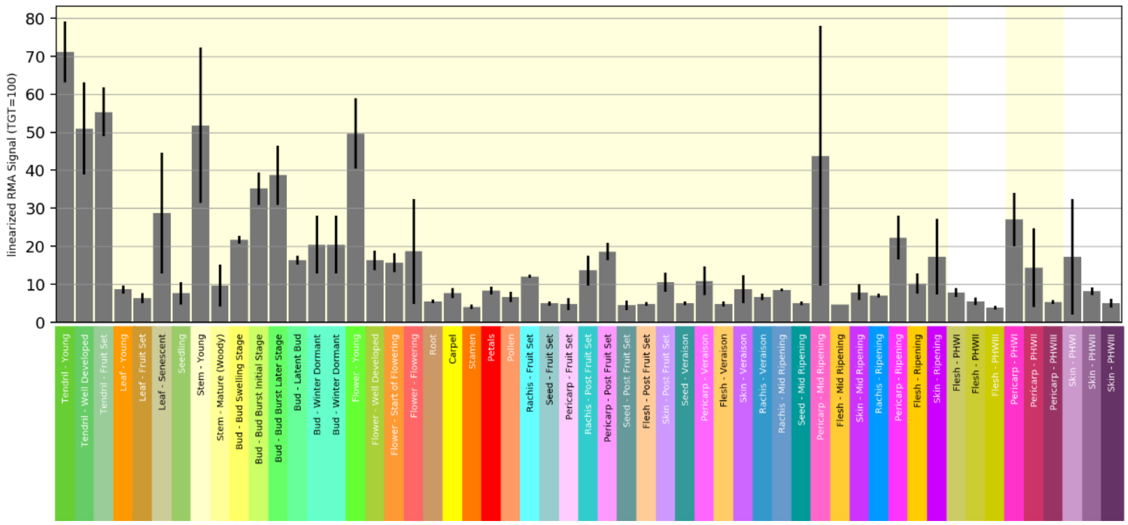

Additional file 10 Figure S1. Expression of the *AmCYC* ortholog *Vitis vinifera GSVIVT01036449001* represented by transcript VIT_14s0083g00150.

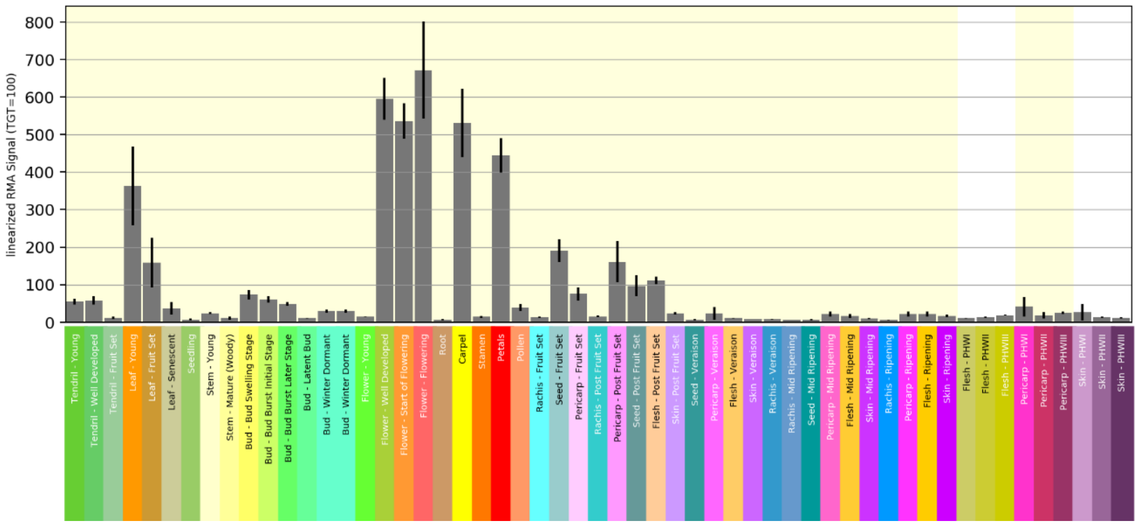

Additional file 10 Figure S2. Expression of the *AmRAD* ortholog *Vitis vinifera GSVIVT01031975001* represented by transcript VIT_03s0063g02620.

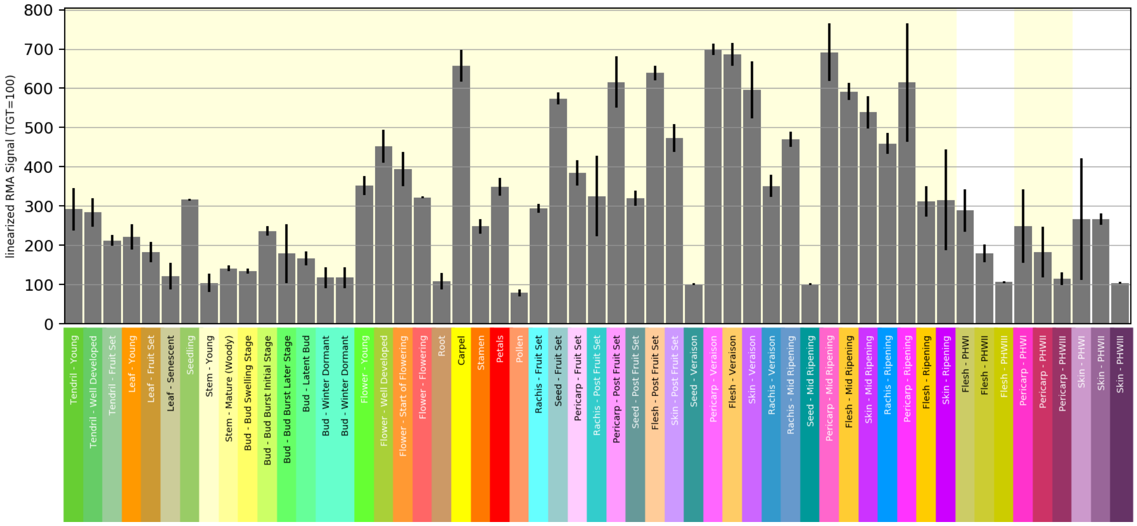

Additional file 10 Figure S3. Expression of the *AmDIV* ortholog *Vitis vinifera DIVlike8* represented by transcript VIT_06s0004g06100.

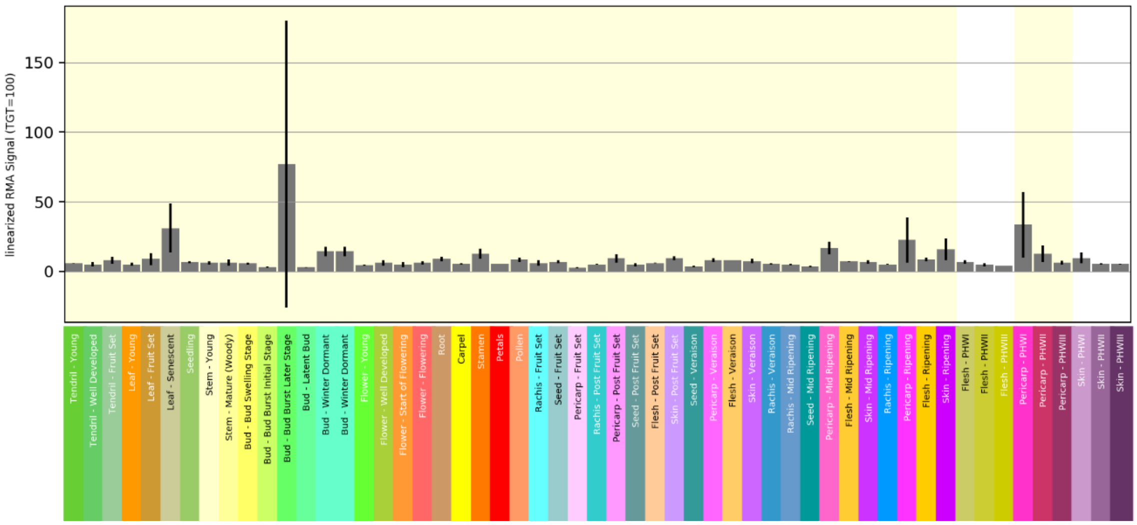

Additional file 10 Figure S4. Expression of the *SlDIVlike5* ortholog/close paralog *Vitis vinifera DIVlike12* represented by transcript VIT_18s0001g02210.

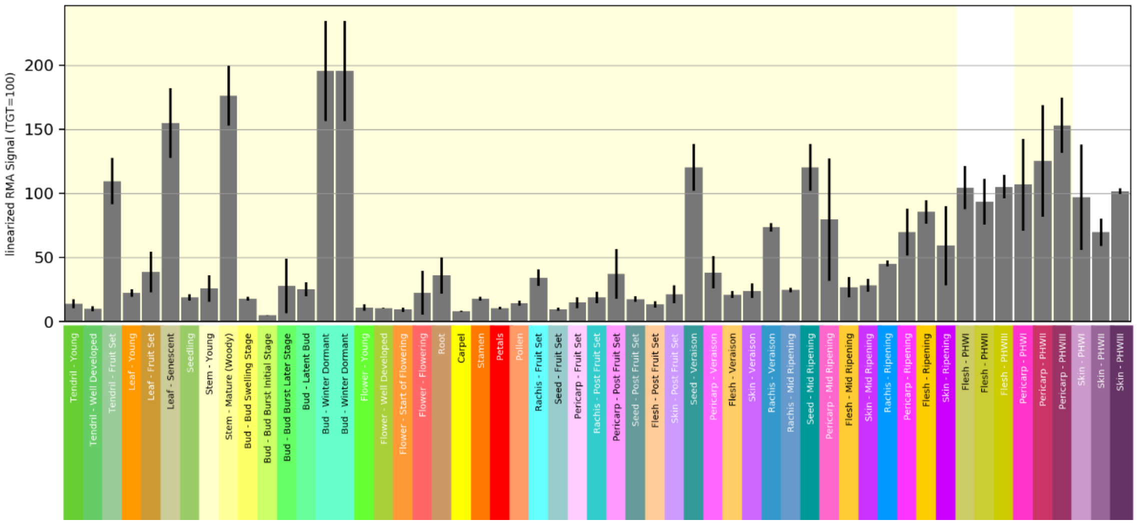

Additional file 10 Figure S5. Expression of the *SlDIVlike5* ortholog/close paralog *Vitis vinifera DIVlike7* represented by transcript VIT_06s0080g00790.

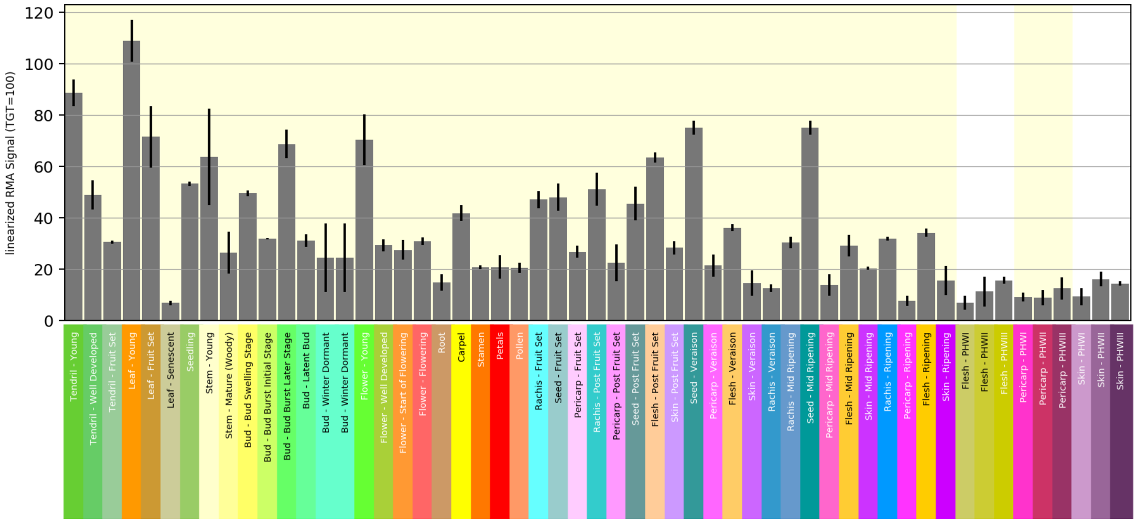

Additional file 10 Figure S6. Expression of the *AmDRIF1* ortholog *Vitis vinifera DRIFlike2* represented by transcript VIT_00s0174g00170 (has multiple transcripts in the expression browser).

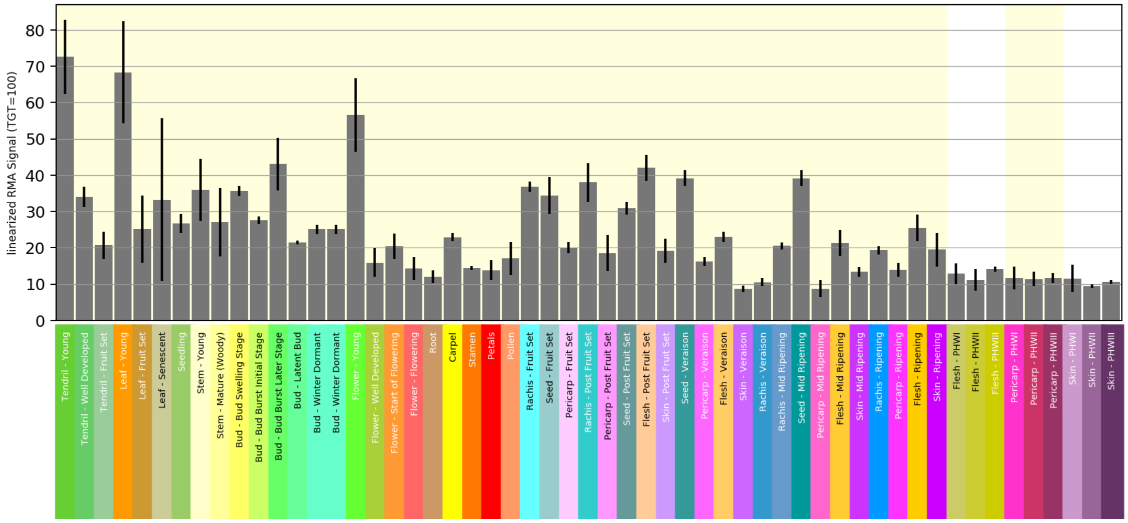

Additional file 10 Figure S7. Expression of the *AmDRIF1* ortholog *Vitis vinifera DRIFlike2* represented by VIT_00s0174g00180 (has multiple transcripts in the expression browser).

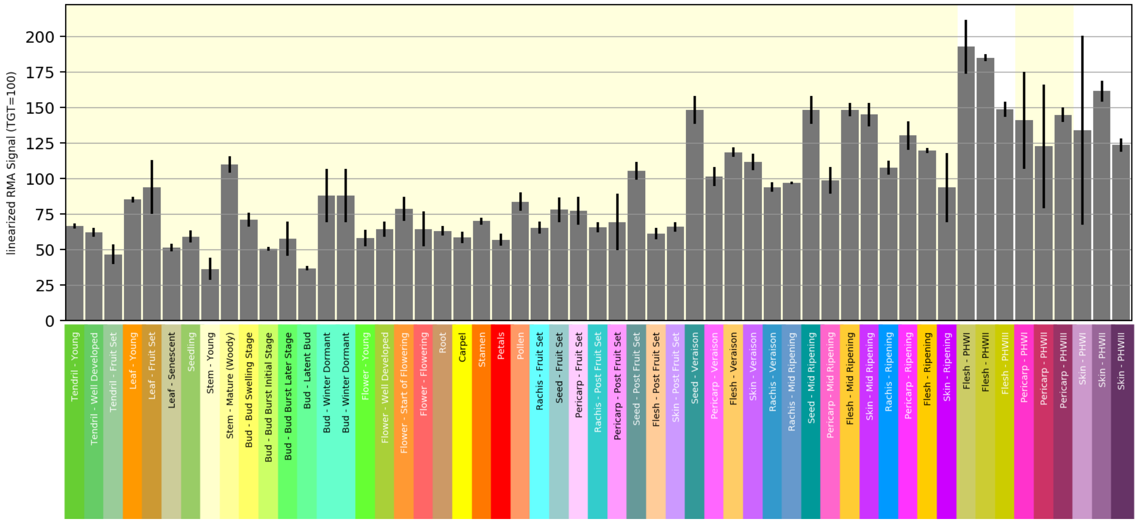

Additional file 10 Figure S8. Expression of the *AmDRIF2* ortholog *Vitis vinifera DRIFlike1* represented by transcript VIT_00s0226g00040.

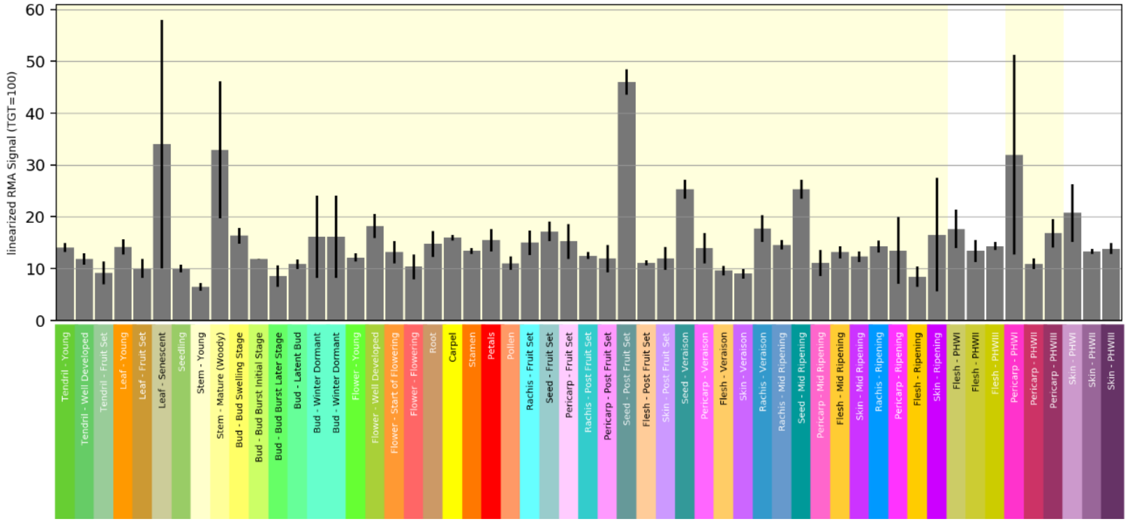

Additional file 10 Figure S9. Expression of the *AmDRIF2* ortholog *Vitis vinifera DRIFlike3* represented by transcript VIT_00s0226g00140 (has multiple transcripts in the expression browser).

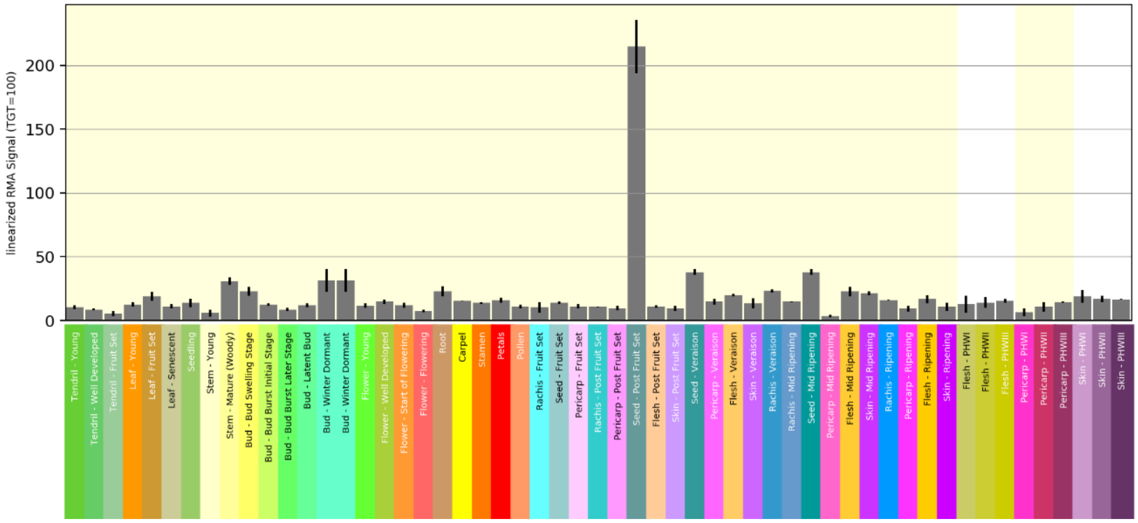

Additional file 10 Figure S10. Expression of the *AmDRIF2* ortholog *Vitis vinifera DRIFlike3* represented by transcript VIT_00s0226g00110 (has multiple transcripts in the expression browser).

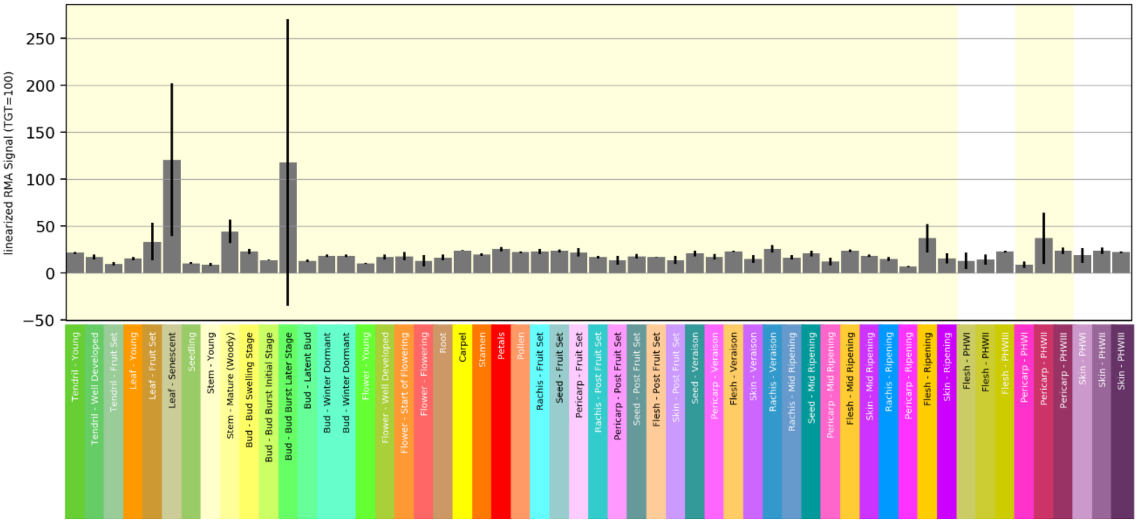

Additional file 10 Figure S11. Expression of the *AmDRIF2* ortholog *Vitis vinifera DRIFlike3* represented by transcript VIT_00s0226g00150 (has multiple transcripts in the expression browser).

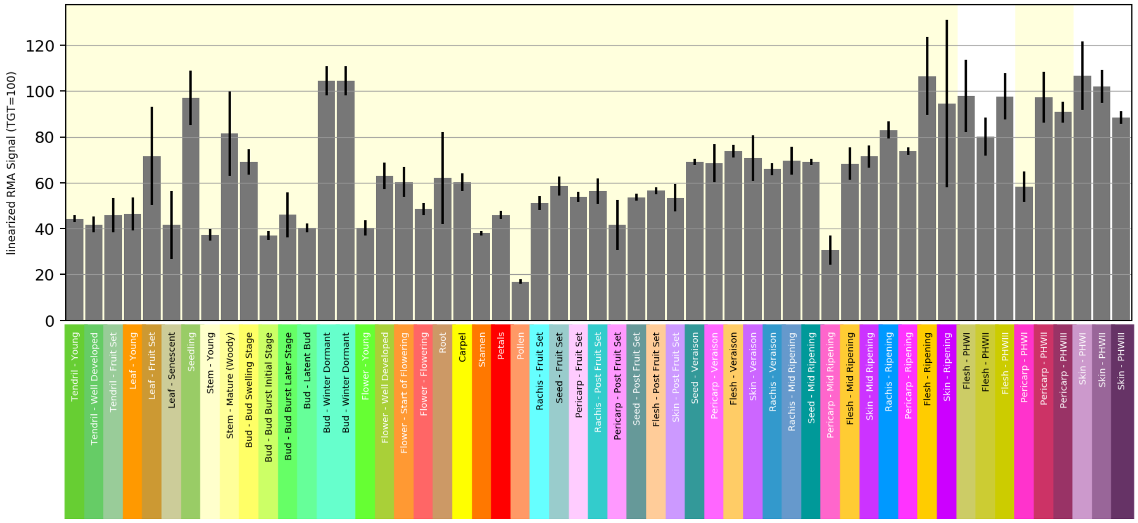

Additional file 10 Figure S12. Expression of the *SlFSB1* ortholog *Vitis vinifera DRIFlike4* represented by transcript VIT_01s0010g01590.
