# Supplementary material for: A CYC–RAD–DIV–DRIF interaction likely pre-dates the origin of floral monosymmetry in Lamiales
Source: EvoDevo. 2022 Jan 29;13:3. doi: 10.1186/s13227-021-00187-w (PMC8801154; doi:10.1186/s13227-021-00187-w)
Supplement: Supplementary file 11 — Additional file 11: Figs. S1–S14. Expression the orthologs of AmCYC, AmRAD, AmDIV, AmDRIF1/2, SlMYBI (SlDIVlike5), and SlFSB1 (SlDRIF5) in Oryza sativa. Images are from bar.utoronto.ca. Expression data for one of the AmRAD orthologs Os05g50350 was not available. [file 13227_2021_187_MOESM11_ESM.pdf]

Additional file 11 Fig. S1–S14. Expression the orthologs of *AmCYC*, *AmRAD*, *AmDIV*, *AmDRIF1/2*, *SlMYBI* (*SlDIVlike5*), and *SlFSB1* (*SlDRIF5*) in *Oryza sativa*. Images are from bar.utoronto.ca. Expression data for one of the *AmRAD* orthologs Os05g50350 was not available.

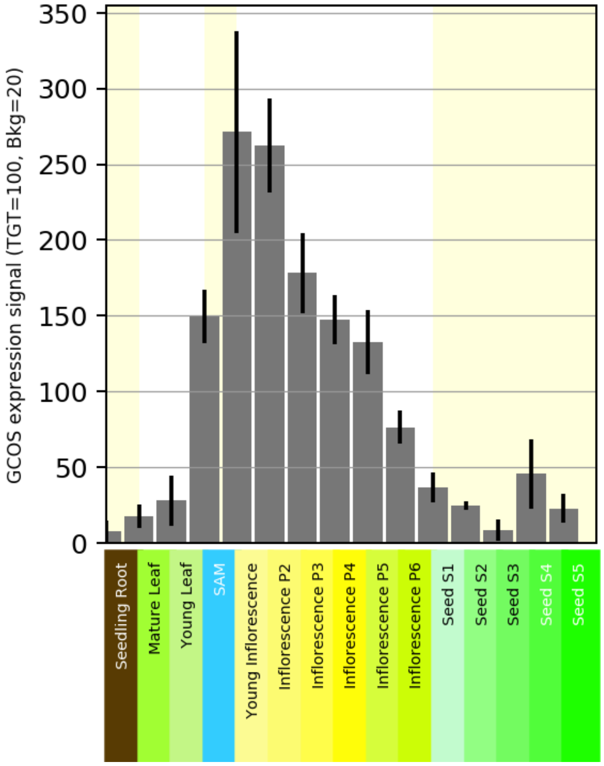

Additional file 11 Figure S1. Expression of the *AmCYC* ortholog of *Oryza sativa* Os03g49880.

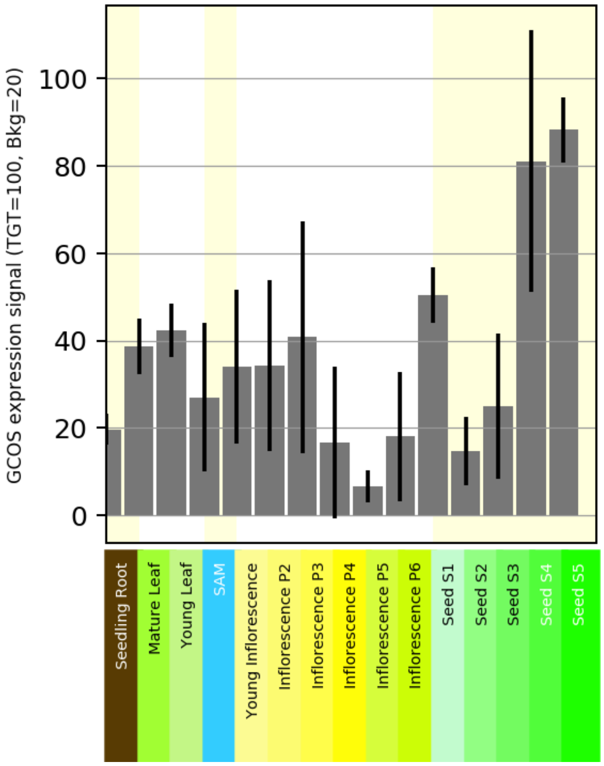

Additional file 11 Figure S2. Expression of the *AmRAD* ortholog of *Oryza sativa* Os12g33950.

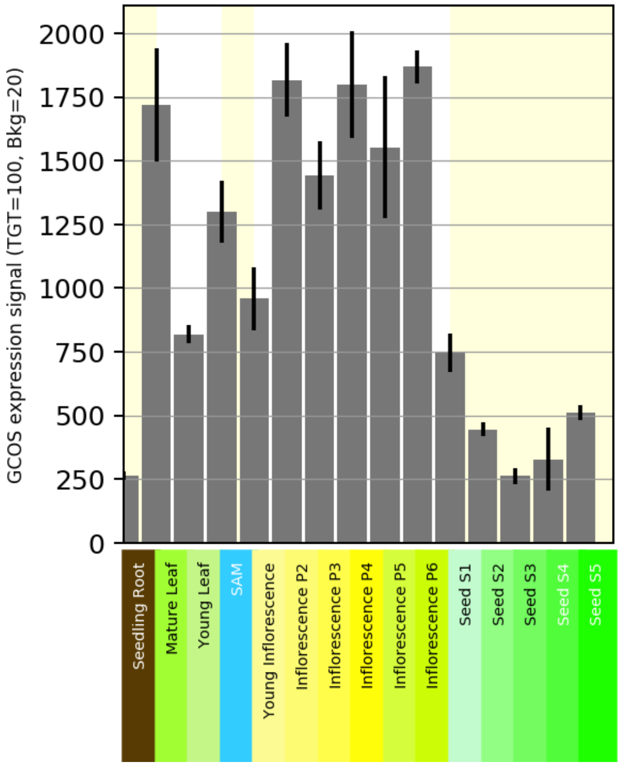

Additional file 11 Figure S3. Expression of the *AmRAD* ortholog of *Oryza sativa* Os02g47744.

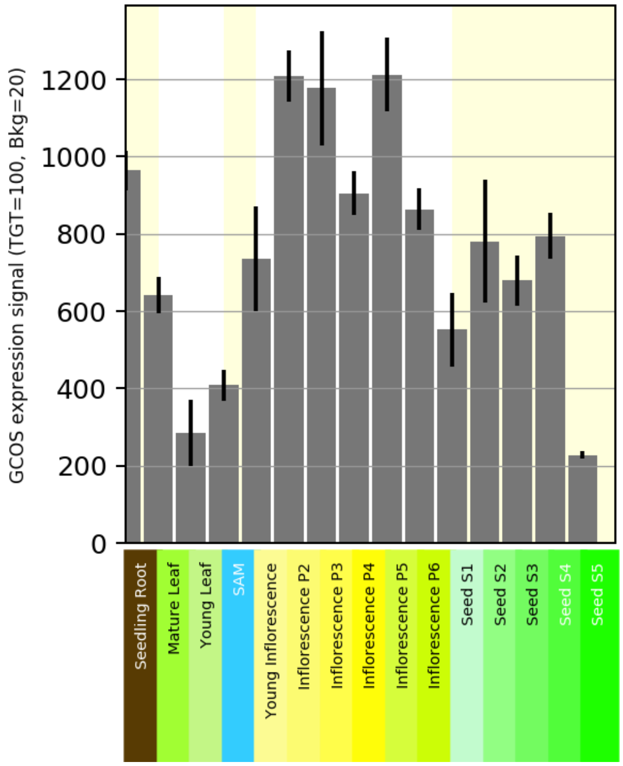

Additional file 11 Figure S4. Expression of the *AmDIV* ortholog *Oryza sativa DIV-like3* (Os01g63460).

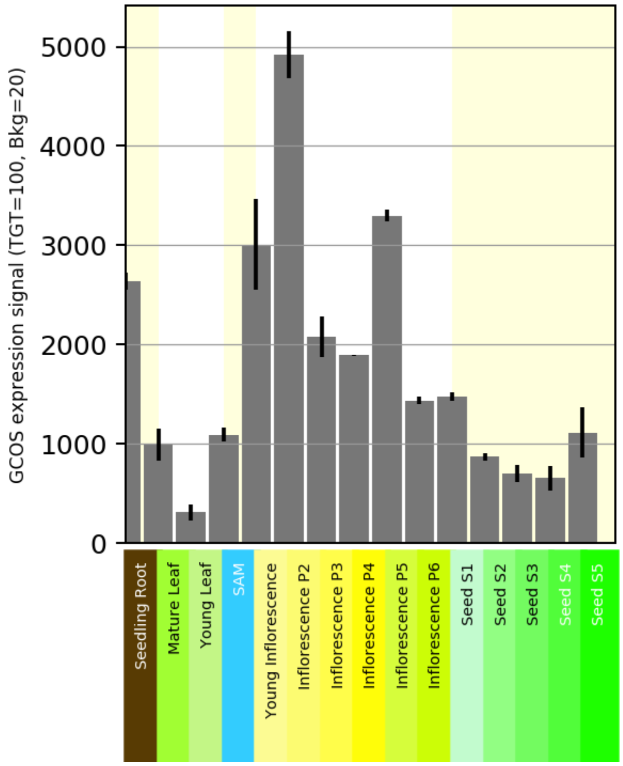

Additional file 11 Figure S5. Expression of the *AmDIV* ortholog *Oryza sativa DIV-like4* (Os05g37730).

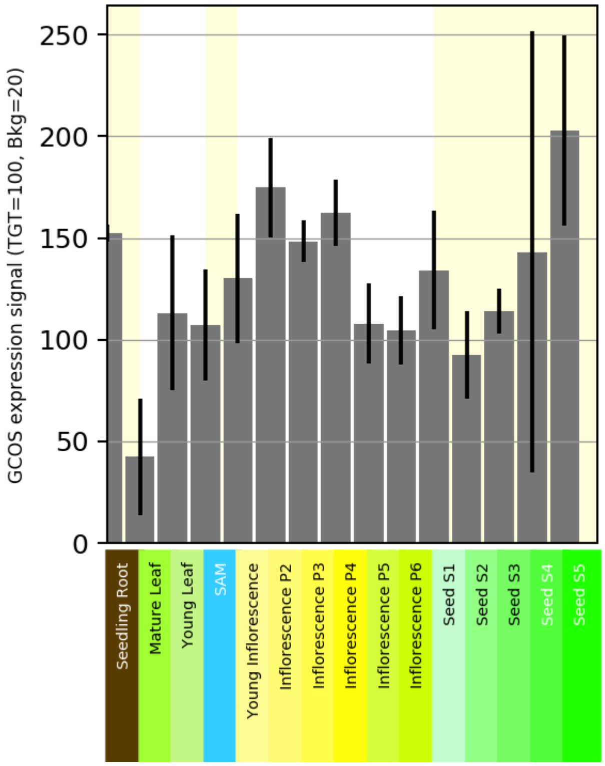

Additional file 11 Figure S6. Expression of the *AmDIV* ortholog *Oryza sativa DIV-like5* (Os01g04930).

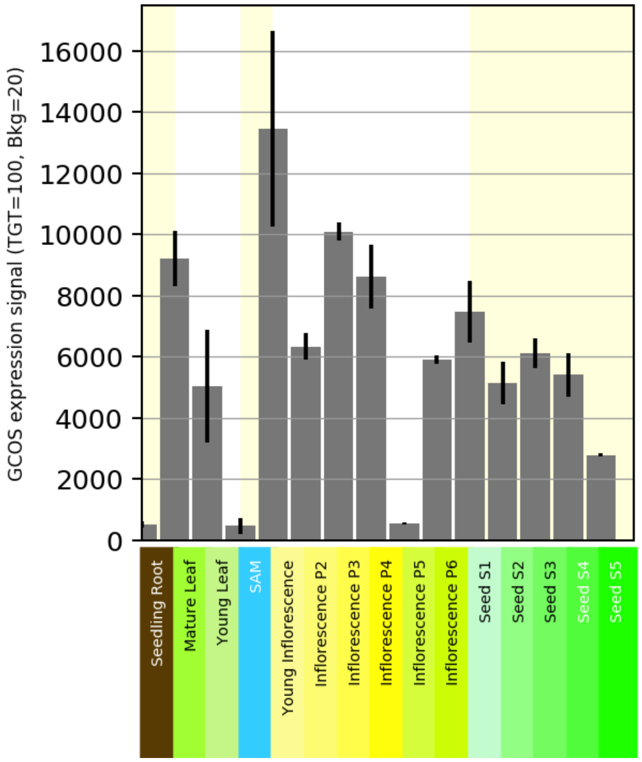

Additional file 11 Figure S7. Expression of the *SlDIVlike5* (*SlMYBI*) ortholog/close paralog *Oryza sativa DIV-like1* (Os01g64360).

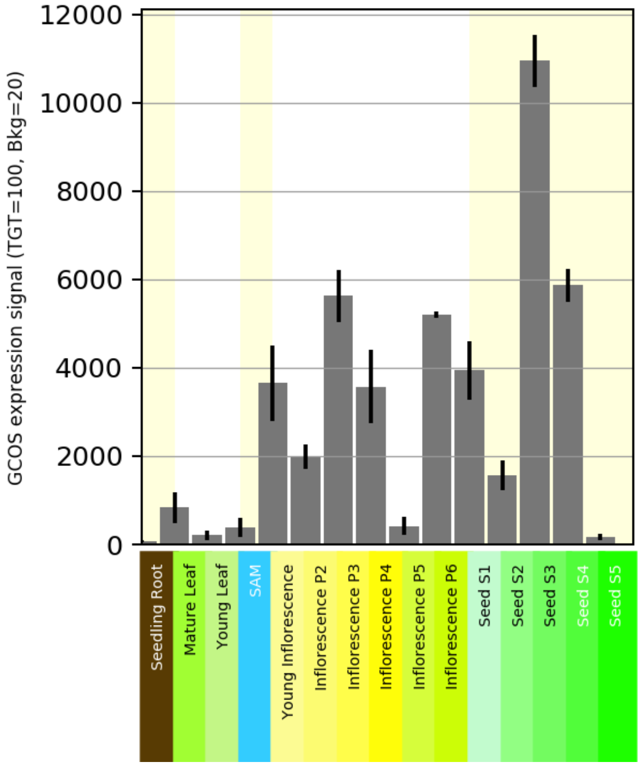

Additional file 11 Figure S8. Expression of the *SlDIVlike5* (*SlMYBI*) ortholog/close paralog *Oryza sativa DIV-like2* (Os05g37060).

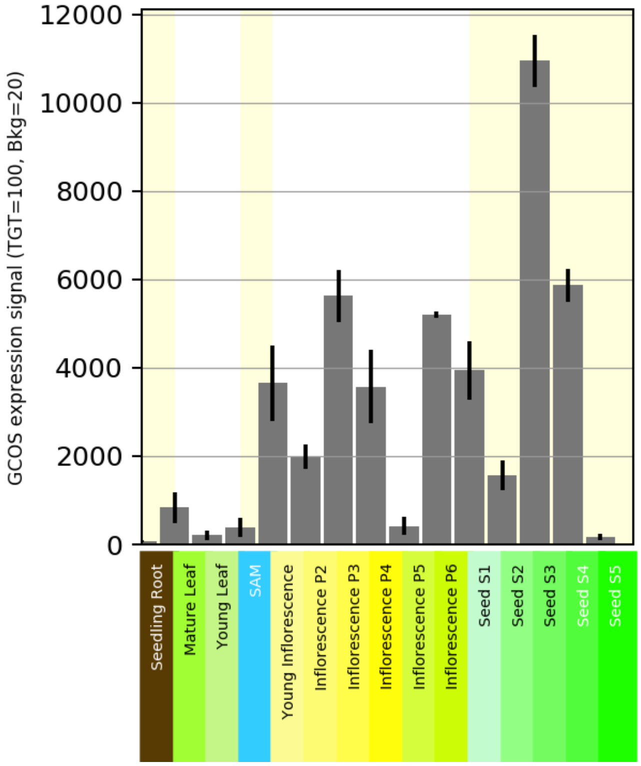

Additional file 11 Figure S9. Expression of the *AmDRIF1/2* ortholog *Oryza sativa DRIFlike1* (Os04g41830).

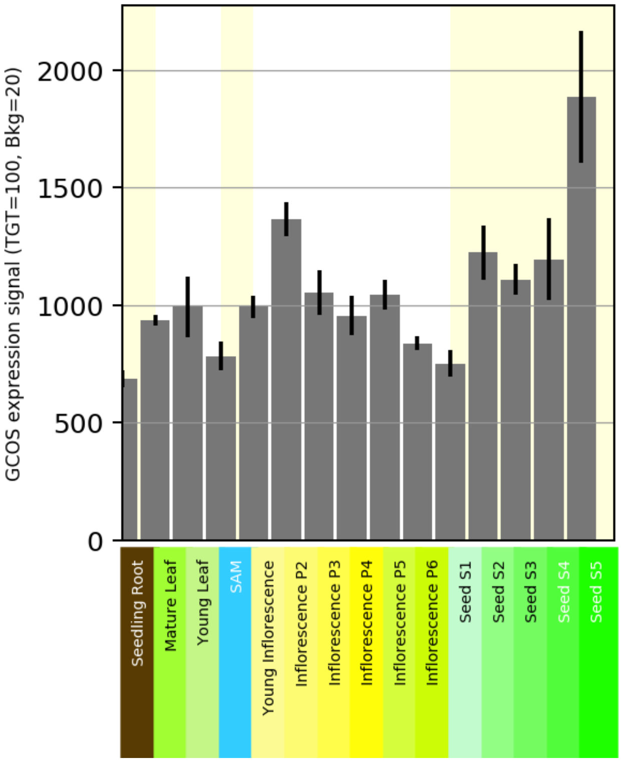

Additional file 11 Figure S10. Expression of the *AmDRIF1/2* ortholog *Oryza sativa DRIFlike2* (Os05g24000).

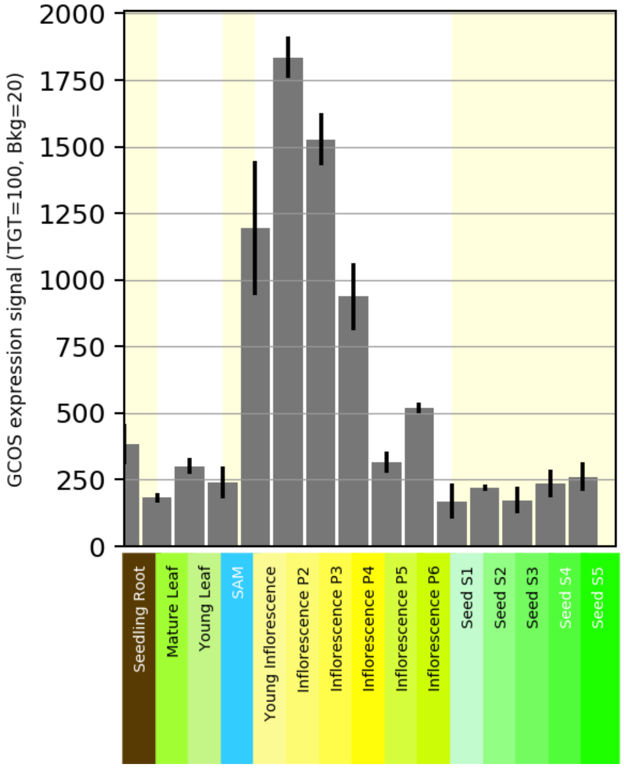

Additional file 11 Figure S11. Expression of the *SlDRIF5* (*SlFSB1*) ortholog *Oryza sativa DRIFlike3* (Os02g42020).

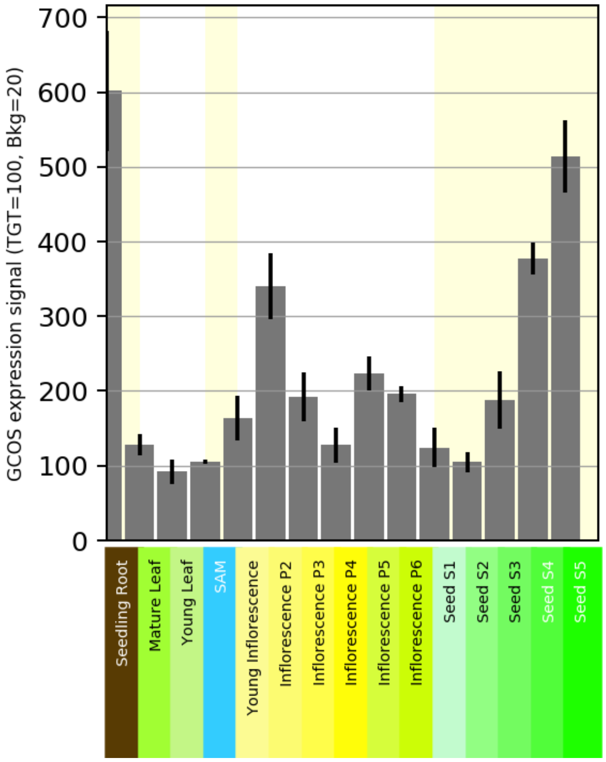

Additional file 11 Figure S12. Expression of the *SlDRIF5* (*SlFSB1*) ortholog *Oryza sativa DRIFlike4* (Os04g44210).

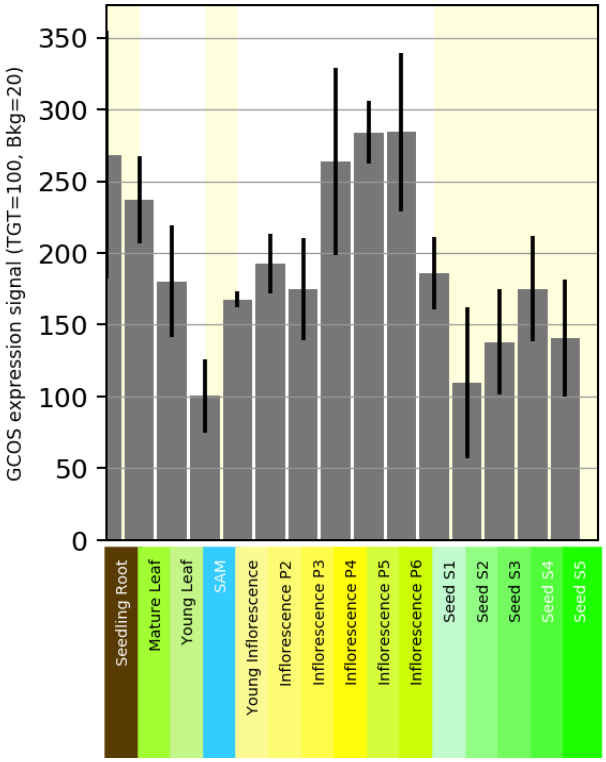

Additional file 11 Figure S13. Expression of the *SlDRIF5* (*SlFSB1*) ortholog *Oryza sativa DRIFlike5* (Os04g57700).

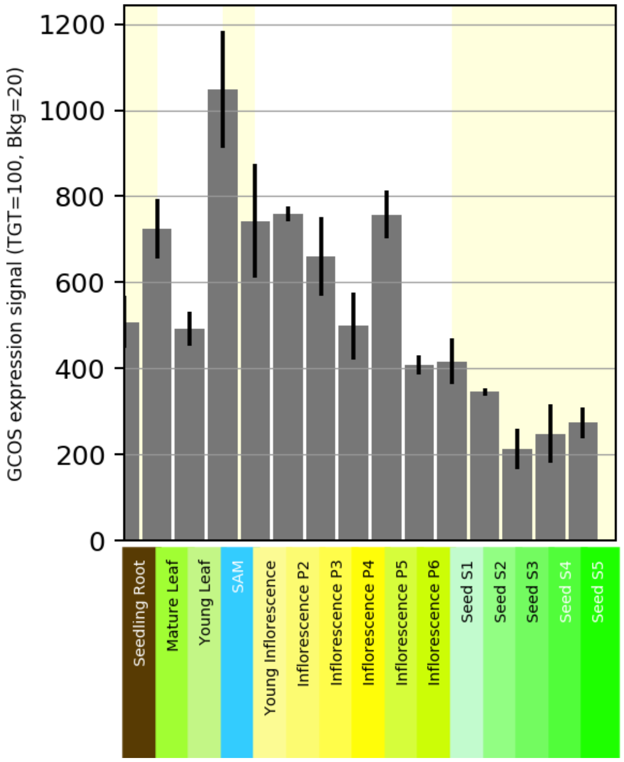

Additional file 11 Figure S14. Expression of the *SlDRIF5* (*SlFSB1*) ortholog *Oryza sativa DRIFlike6* (Os08g01080).
